# CCN2-induced lymphangiogenesis is mediated by the integrin αvβ5–ERK pathway and regulated by DUSP6

**DOI:** 10.1038/s41598-022-04988-4

**Published:** 2022-01-18

**Authors:** Shiho Hashiguchi, Tomoko Tanaka, Ryosuke Mano, Seiji Kondo, Shohta Kodama

**Affiliations:** 1 Department of Oral Surgery, Faculty of Medicine, Fukuoka University, Fukuoka, Japan; 2 Department of Regenerative Medicine and Transplantation, Faculty of Medicine, Fukuoka University, Fukuoka, Japan

**Keywords:** Cell biology, Cell signalling, Extracellular signalling molecules

## Abstract

Lymphangiogenesis is essential for the development of the lymphatic system and is important for physiological processes such as homeostasis, metabolism and immunity. Cellular communication network factor 2 (CCN2, also known as CTGF), is a modular and matricellular protein and a well-known angiogenic factor in physiological and pathological angiogenesis. However, its roles in lymphangiogenesis and intracellular signaling in lymphatic endothelial cells (LECs) remain unclear. Here, we investigated the effects of CCN2 on lymphangiogenesis. In in vivo Matrigel plug assays, exogenous CCN2 increased the number of Podoplanin-positive vessels. Subsequently, we found that CCN2 induced phosphorylation of ERK in primary cultured LECs, which was almost completely inhibited by the blockade of integrin αvβ5 and partially decreased by the blockade of integrin αvβ3. CCN2 promoted direct binding of ERK to dual-specific phosphatase 6 (DUSP6), which regulated the activation of excess ERK by dephosphorylating ERK. In vitro, CCN2 promoted tube formation in LECs, while suppression of *Dusp6* further increased tube formation. In vivo, immunohistochemistry also detected ERK phosphorylation and DUSP6 expression in Podoplanin-positive cells on CCN2-supplemented Matrigel. These results indicated that CCN2 promotes lymphangiogenesis by enhancing integrin αvβ5-mediated phosphorylation of ERK and demonstrated that DUSP6 is a negative regulator of excessive lymphangiogenesis by CCN2.

## Introduction

Lymphatic vessels regulate tissue fluid homeostasis, immune cell trafficking, and absorption of dietary fats^1^. Formation of new lymphatic vessels, or lymphangiogenesis, plays an important role in normal physiological processes during development as well as in a number of pathological processes like inflammation, wound healing, and tumor metastasis^2^. Impairment of lymphatic function results in various diseases that are characterized by the inadequate transport of interstitial fluid, edema, impaired immunity, and fibrosis^3,4^. Abnormal lymphatic growth around tumors facilitate metastatic spread of malignant cells^5^. Vascular endothelial growth factor-C (VEGF-C)-Vascular endothelial growth factor receptor-3 (VEGFR-3) signaling is crucial for the development of lymphatic vessels^6,7^. Several angiogenic factors are also involved in growth of lymphatic vessels, such as the Tie /angiopoietin system, neuropilin-2 and integrin α9, which regulate lymphangiogenesis^8–10^.

Cellular communication network factor 2 (CCN2), also known as connective tissue growth factor (CTGF), was originally isolated as a platelet-derived growth factor (PDGF)-like factor in human umbilical vein endothelial cell (HUVEC)^11^. CCN2 is a member of the CCN family of modular matricellular proteins, which contains six family members (CCN1-6)^12^. CCN2 is a multifunctional molecule, with roles in angiogenesis, development, tumorigenesis, fibrotic disease and wound healing^13^. CCN family proteins contains four structural domains: the IGF binding domain, the von Willebrand factor C repeat domain, the thrombospondin type 1 (TSP) domain and the cysteine knot C-terminal domain. Each module interacts with other cell surface receptors, growth factors, structural matrix proteins^12^. In particular, integrins are involved in many CCN2-mediated biological processes; The TSP domain in CCN2 interacts with integrin α6β1^14^, and the C-terminal motif of CCN2 interacts with integrins αvβ3, α5β1^15,16^. CCN2-mediated cell adhesion via integrin αvβ3 or α6β1 requires cell surface heparan sulfate proteoglycans (HSPGs), which act as co-receptors for integrins^17^. By binding to cell surface receptors, CCN2 can alter various intracellular signaling pathways, including the mitogen-activated protein kinase (MAPK) pathway^18–20^, WNT pathway^21^, NFκB pathway^22^ and others. Among them, the extracellular signal-regulated kinase (ERK)-dependent pathway provides a variety of CCN2-mediated functions, including angiogenesis, fibrosis, inflammation, bone metabolism, and tumorigenesis^18,19,23–25^. Activation of ERK by CCN2 can be transient or persistent^17^, depending on the cell type and microenvironment, but when and how ERK is inactivated after CCN2 stimulation is controversial. The dual-specific phosphatase (DUSP) family phosphatases are the largest group of protein phosphatases that specifically regulate MAPK activity in mammalian cells^26^. The relationship between DUSPs and CCN2 is unknown.

The angiogenesis-promoting effect of CCN2 has been shown in vascular endothelial cell culture experiments and animal studies. CCN2 directly binds to integrin αvβ3 and promotes endothelial cell adhesion, migration, and survival^27^. In vivo, it has been shown to promote angiogenesis in CAM assays, collagen pellet implantation, and Matrigel plug assays^28,29^. CCN2 is highly expressed in endothelial cells during development, and *Ccn2* knockout mice have impaired angiogenesis in the growth plate, resulting in skeletal abnormalities and perinatal lethality^30^. Normal basement membrane formation is also impaired in *Ccn2* knockout mice^31^. In breast and prostate cancers, tumor cells and tumor stroma secrete CCN2, which promotes endothelial cell migration and induces tumor angiogenesis^32,33^. CCN2 binds to integrin αvβ3 on endothelial cells and activates the p38 MAPK, ERK, and Jun N-terminal kinase (JNK) MAPK signaling pathways^34,35^. CCN2 is a complex regulator of angiogenesis because it not only directly regulates endothelial cell function, but also controls the production and activity of other angiogenic molecules (e.g., bFGF and VEGF) and molecules that affect extracellular matrix (ECM) integrity and stability (e.g., collagen, matrix metalloproteinases (MMPs), and tissue inhibitor of MMPs)^36^. Lymphatic vessels differ significantly from blood vessels in their specific structure and function and in molecular mechanisms that regulate their development and growth^2^, and it is still not well understood how CCN2 acts in lymphangiogenesis.

Previous studies in renal, peritoneal dialysis-related and liver fibrosis mouse models showed that CCN2 enhanced lymphangiogenesis^37,38^. Furthermore, CCN2 is involved in fibrosis-associated renal lymphangiogenesis through its interaction with VEGF-C^37,39^. These reports show that CCN2 is closely associated with lymphangiogenesis in fibrous diseases. However, it has not been elucidated how CCN2 acts on physiological lymphangiogenesis. In this study, we investigated the effect of CCN2 on lymphangiogenesis in vivo and the downstream signaling of CCN2 in primary cultured lymphatic endothelial cells (LECs).

## Results

### CCN2 promotes lymphangiogenesis in in vivo Matrigel plug assay

**Figure 1:**
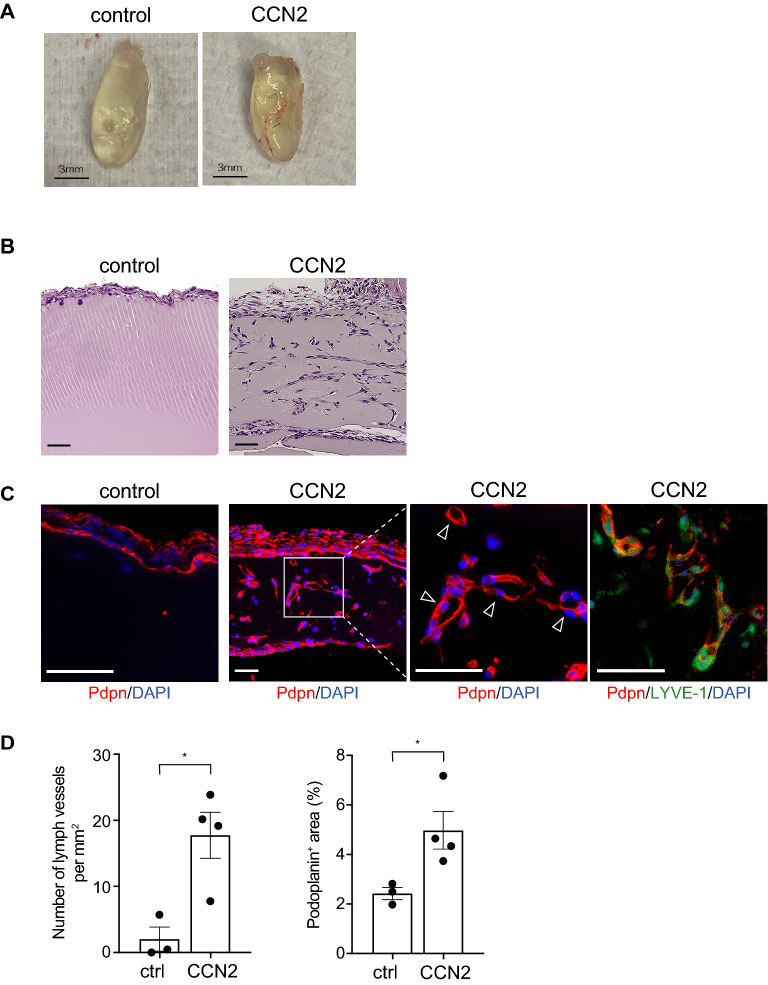
CCN2 enhances lymphangiogenesis in *in vivo* Matrigel plug assay. Matrigel and recombinant CCN2 were mixed and injected subcutaneously back of the mice. Control mice were injected with Matrigel and PBS mixture. (**A**) Images of Matrigel removed 7 days after injection. Scale bars: 3 mm. (**B**) HE-stained sections of Matrigel. Bars: 50 µm. (**C**) Immunostaining of Podoplanin and LYVE-1. Arrowheads indicate Podoplanin-positive vessels in the CCN2 gel. Bars: 50 µm. (**D**) Number of Podoplanin-positive vessels per mm^2^ and the ratio of the Podoplanin-positive area to the whole area. Student *t* test, *p < 0.05.

To examine the role of CCN2 in lymphangiogenesis in vivo, the Matrigel plug assay was performed. Recombinant CCN2 was mixed with Matrigel and subcutaneously injected onto the backs of mice. At 7 days after injection, blood vessels were macroscopically observed on the surface of plugs in the CCN2 group mice, but not in the control group (Fig. 1A). Histological analyses revealed that the cells in the CCN2 gel were mostly Podoplanin-positive, and tubular structures were observed inside of the gel (Fig. 1B,C). Double immunohistochemical staining showed Lymphatic vessel endothelial hyaluronan receptor 1 (LYVE-1) expression was detected in Podoplanin positive cells (Fig. 1C). The number of Podoplanin-positive vessels and percentage of Podoplanin-positive areas were increased in CCN2-supplemented gels compared with controls, which suggests that CCN2 positively modulated lymphangiogenesis (Fig. 1D). CCN2 also increased the number of CD31-positive cavities and β3-Tubulin-positive peripheral nerves in the CCN2 plugs (Supplementary Fig. S1).

### CCN2 increases the gene expression levels of LEC markers in primary cultured LECs

**Figure 2:**
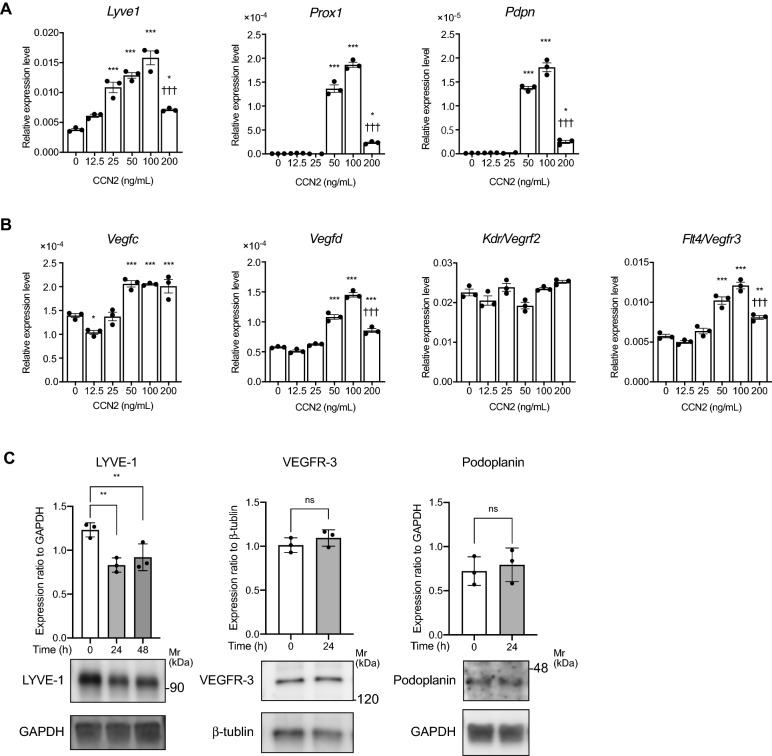
Expression levels of lymphatic endothelial markers in CCN2-treated LECs. Mouse primary cultured LECs were treated with 0, 12.5, 25, 50, 100 or 200 ng/mL CCN2 for 3 h. (**A**) mRNA expression levels of *Lyve1*, *Podoplanin* and *Prox1* were analyzed by quantitative RT-PCR. (**B**) *Vegfc, Vegfd, Kdr* (encoding VEGFR2) and *Flt4* (encoding VEGFR3) mRNA levels were analyzed by quantitative RT-PCR. *Actb* was used as an internal control, and gene expression levels were expressed relative to *Actb* mRNA. One-way ANOVA, *p < 0.05, **p < 0.01, ***p < 0.001 versus 0 ng/mL CCN2. ^†††^*p* < 0.01 versus 100 ng/mL CCN2. (**C**) LECs were treated with 100 ng/ml CCN2 for 24 h, and protein expression levels of LYVE-1, VEGFR3, and Podoplanin were detected by Western blotting. One-way ANOVA, **p < 0.01 versus 0 h. Student *t* test, ns; not significant.

In order to clarify the signaling pathway by which CCN2 is activated in lymphatic endothelium, we performed experiments using primary cultures of lymphatic endothelial cells. The expression levels of LEC markers and vascular endothelial markers in LECs were examined by quantitative RT-PCR, and LECs expressed higher levels of *Lyve1* and *Vegfr3* and lower levels of *Pecam1* compared to vascular endothelial cells (Supplementary Fig. S2A). The results of cellular immunostaining showed that the LYVE-1 positivity rate of LECs was 95% (Supplementary Fig. S2B). Next, LECs were stimulated with 0, 12.5, 25, 50, 100 or 200 ng/mL of CCN2 for 3 h, and the expression levels of the LEC markers *Lyve1*, *Podoplanin* (*Pdpn)* and *prospero homeobox 1 (Prox1)* were analyzed by quantitative RT-PCR. The expression levels of *Lyve1*, *Pdpn* and *Prox1* were increased by CCN2 in a dose-dependent manner up to 100 ng/mL; at 200 ng/mL, their expression levels dramatically decreased compared with levels at 100 ng/mL (Fig. 2A). We further found that CCN2 increased the expression levels of the lymphangiogenic factors *Vegfc*, *Vegfd* and *Flt4* (encording VEGFR3) but did not affect *Kdr* (encording VEGFR2) expression (Fig. 2B). The expression levels of LYVE-1, Podoplanin, PROX1, and VEGFR3 proteins in LECs stimulated with CCN2 for 0, 24, and 48 h were analyzed by Western blotting. Podoplanin and VEGFR3 expression levels were not changed by CCN2 stimulation. PROX1 was undetectable. Real-time PCR results showed that *Lyve1* mRNA was upregulated by CCN2, whereas LYVE-1 protein expression was downregulated by CCN2 (Fig. 2C). Phosphorylation of the receptor tyrosine kinases (RTKs) VEGFR2 and VEGFR3 were investigated using a phospho-RTK array. Our results showed that VEGFR2 and VEGFR3 phosphorylations were unchanged by CCN2 (Supplementary Fig. S3A,B). In contrast, phosphorylation levels of the RTKs Epidermal growth factor receptor (EGFR) and Erythropoietin-producing hepatocellular receptor B4 (EphB4) were increased by CCN2, while phosphorylation of Epidermal growth factor receptor 4 (ErbB4) and Platelet-derived growth factor receptor alpha (PDGFRα) were decreased compared with controls (*p* < 0.001) (Supplementary Fig. S3A,B).

### CCN2 enhances the phosphorylation of ERK via integrin αvβ5 in LECs

**Figure 3:**
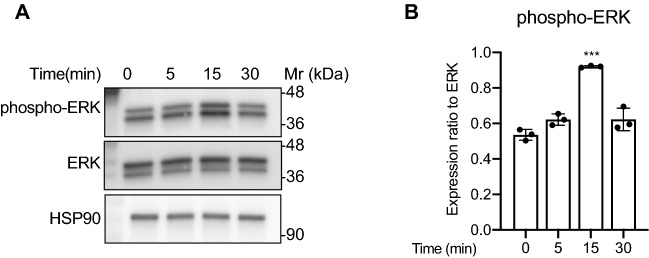
Phosphorylation of ERK is increased by CCN2. (**A**) LECs were stimulated with 100 ng/mL CCN2 for 0, 5, 15 or 30 min, and phosphorylated ERK, total ERK and HSP90 were detected by Western blotting. (**B**) Quantification of protein bands from (**A**); phospho-ERK level was normalized with total ERK. One-way ANOVA, ***p < 0.001 versus 0 min.

To determine the intracellular signaling downstream of CCN2, we next analyzed the expression of various phosphorylated proteins that are known to be activated by CCN2 stimulation^12^. While the phosphorylation levels of p38 MAPK, JNK, AKT, NFκB and Smad2, -3, and -1/5 were unchanged in response to CCN2 (Supplementary Fig. S4A-C), phospho-ERK was significantly increased in response to CCN2 (*p* < 0.001) (Fig. 3A,B).

**Figure 4:**
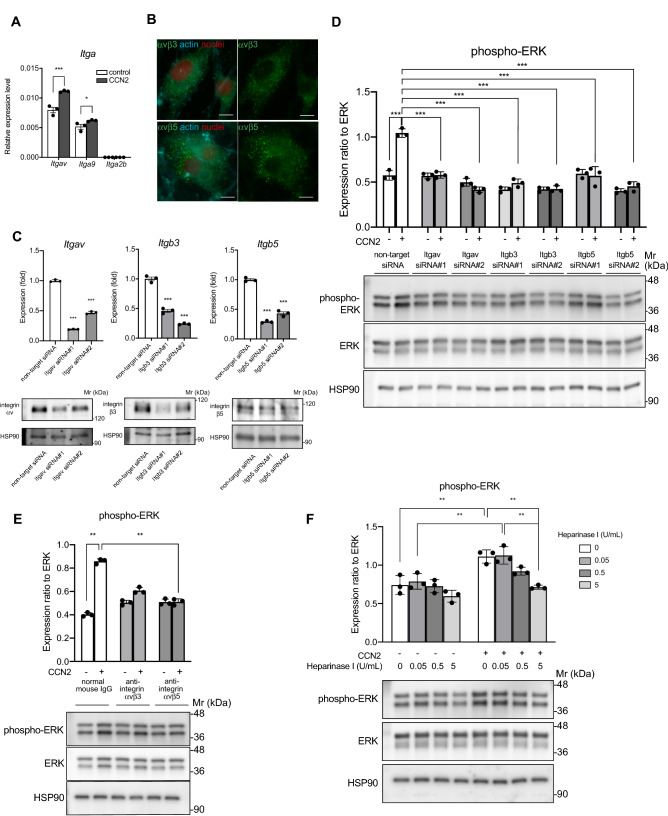
CCN2 induces phospho-ERK levels via integrin αvβ5. (**A**) LECs were treated with or without 100 ng/mL CCN2, and mRNA expression levels of *Itgav, Itga9*, and *Itga2b* were evaluated by quantitative RT-PCR. Gene expression was normalized to levels of *Actb* mRNA. Two-way ANOVA, * p < 0.05, *** p < 0.001. (**B**) LECs were immunostained using anti-integrin αvβ3 or αvβ5 antibodies. Scale bars: 10 µm. (**C**) Suppression of integrins αv, β3 and β5 by siRNA. mRNA expression levels (upper) and protein levels (lower) in LECs transfected with non-target siRNA or integrins αv, β3 and β5 siRNA. One-way ANOVA, *** p < 0.001 versus non-target siRNA. (**D**)Western blot in LECs transfected with siRNA for *Itgav, b3* and *b5* and treated with CCN2. Phospho-ERK levels were quantified and normalized with total ERK expression levels, Two-way ANOVA, ***p < 0.001. ns; not significant. (**E**) The effects of integrin αvβ3 or αvβ5 neutralizing antibody on CCN2-induced ERK phosphorylation were examined by Western blotting, and the values of phospho-ERK were normalized by the expression level of total ERK. One-way ANOVA, ** p < 0.01 versus control LEC. (**F**) Phosphorylation of ERK in LECs pretreated with 0, 0.05, 0.5, or 5 U/mL heparinase I and stimulated with 100 ng/ml CCN2 for 10 min. Two-way ANOVA, ** p < 0.01.

Although VEGFRs are upstream of ERK^40,41^, our RTK assay results showed that VEGFR2 and VEGFR3 were not activated by CCN2 in LECs (Supplementary Fig. S3B). Previous studies showed that CCN2 promotes vascular endothelial cell growth, migration, adhesion, and survival partly through its interactions with integrins^42^. Therefore, we next investigated whether CCN2-mediated effects on ERK may involve its interaction with integrins. We first examined the expression levels of *Itgav, Itga9, Itga2b* mRNA in primary LECs treated with CCN2. *Itgav, Itga9* were predominantly expressed in LECs (Fig. 4A). Furthermore, the expression levels of these mRNAs were increased by CCN2 stimulation (Fig. 4A). *Itga2b* were not induced by CCN2 (Fig. 4A). Cellular immunostaining showed that integrins αvβ3 and αvβ5 are expressed in LECs (Fig. 4B). Knockdown of integrins αv, β3 and β5 by siRNA decreased phosphorylation of ERK induced by CCN2 compared with non-target siRNA (*p* < 0.001, *p* < 0.001, *p* < 0.001) (Fig. 4C,D), which suggests that CCN2 increases phospho-ERK via integrins αv, β3, and β5 in LECs. Furthermore, CCN2-induced phospho-ERK was significantly reduced by co-incubation with a function-blocking antibody against integrins αvβ3 and αvβ5 compared with IgG isotype control. In particular, the blockade of integrin αvβ5 almost completely blocked the phosphorylation of ERK by CCN2 (Fig. 4E). These results indicate that CCN2 activates ERK via integrins αvβ3 and αvβ5 in LECs and that integrin αvβ5 is predominant in CCN2 signaling in LECs. In contrast, knockdown of integrin α9 and β1 resulted in a slight increase of CCN2-induced phospho-ERK compared with levels in cells transfected with non-target siRNA (Supplementary Fig. S5A,B). *Itgb2* and *Itgb3* mRNA levels were not changed by suppression of integrin β1, which suggests that integrins β2 and β3 may not compensate for the decrease in integrin β1 (Supplementary Fig. S5C). CCN2-mediated cell adhesion via integrin αvβ3 exhibited the absolute requirement for heparan sulfate proteoglycans (HSPGs), and destruction of cell surface HSPGs with heparinase abrogated cell adhesion to CCN2^17^. To substantiate the finding that HSPGs are required for CCN2-induced ERK activation in LECs, LECs were treated with heparinase I, an enzyme that targets highly sulfated HSPGs. In heparinase I-treated cells, ERK phosphorylation with stimulation of CCN2 was inhibited in a concentration-dependent manner by heparinase I (Fig. 4F). These results show that CCN2 signaling in LECs requires cell surface HSPGs.

### CCN2 enhances interaction of DUSP6 and ERK, and DUSP6 negatively regulates CCN2-mediated lymphangiogenesis

**Figure 5:**
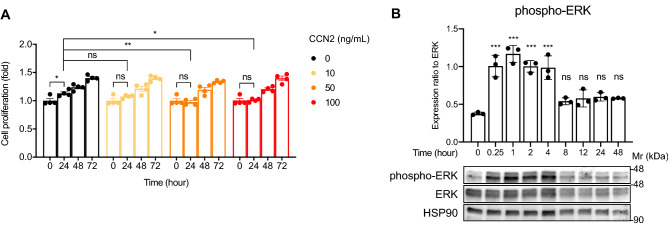
Weak inhibitory effects of CCN2 on the proliferation of LECs and transient phosphorylation of ERK induced by CCN2. (**A**) LECs were cultured in medium containing 0, 10, 50 or 100 ng/mL of CCN2 for 24, 48 or 72 h. Cell proliferation was analyzed by crystal violet staining. Cell proliferation was expressed as the ratio to the value at 0 h. Two-way ANOVA, * p < 0.05, ** p < 0.01. ns; not significant. (**B**) LECs were treated with 100 ng/mL CCN2 for 0, 0.25, 1, 2, 4, 8, 12, 24 or 48 h, and phospho-ERK, ERK and HSP90 were detected by Western blot. Phospho-ERK levels at each time point were normalized to those of total ERK. One-way ANOVA, *** p < 0.001 versus 0 h. ns; not significant versus 0 h.

CCN2 promotes cell proliferation in vascular endothelium^28^. Therefore, we next analyzed the effects of CCN2 on the cell growth of LECs. Control LECs showed cell proliferation after 24 h of incubation (*p* = 0.01), whereas 10, 50 and 100 ng/mL CCN2-stimulated LECs did not show significant proliferation (*p* = 0.22, 0.92 and > 0.99, respectively) (Fig. 5A). In addition, treatment with 50 and 100 ng/mL of CCN2 suppressed cell growth at 24 h compared with vehicle-treated LECs (*p* = 0.003 and 0.02). However, no changes in proliferation were observed at 48 and 72 h of treatment. These results indicate CCN2 has a weak growth inhibitory effect on LECs.

**Figure 6:**
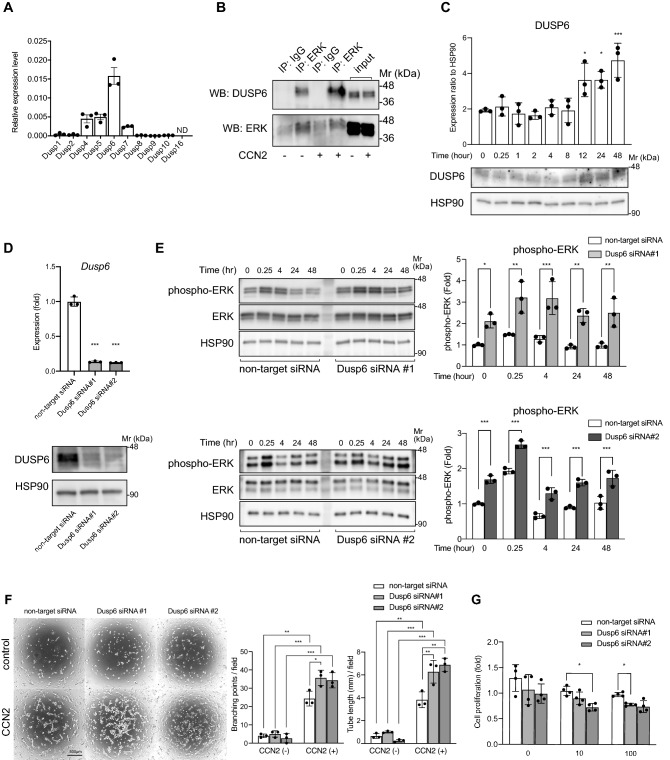
DUSP6 interacts with ERK and suppression of DUSP6 increases phospho-ERK levels and tube formation. (**A**) Expression levels of *Dusp1*, *-2*, *-4*, *-5*, *-6*, *-7*, *-8*, *-9, -10* and *-16* in LECs. ND, not detected. (**B**) Cell lysates from LECs stimulated with 100 ng/mL of CCN2 for 2 h or control cells were immunoprecipitated with anti-ERK antibody (ERK) or normal rabbit IgG (IgG) and analyzed by Western blot with antibodies against DUSP6 or ERK. (**C**) Expression levels of DUSP6 protein in LECs treated with CCN2 for 0, 0.25, 1, 2, 4, 8, 12, 24, and 48 h. (**D**) Suppression of DUSP6 by siRNA. mRNA expression levels (upper) and protein levels (lower) in LECs transfected with non-target siRNA or DUSP6 siRNA. One-way ANOVA, ***p < 0.001 versus non-target siRNA. (**E**) Phospho-ERK levels in LECs transfected with non-target siRNA or DUSP6 siRNA, and treated with 100 ng/mL CCN2 for 0, 0.25, 4, 24 or 48 h. phospho-ERK levels were quantified and normalized with total ERK levels. Two-way ANOVA, *p < 0.05, **p < 0.01, ***p < 0.001, versus non-target siRNA. (**F**) LECs were transfected with siRNA against *Dusp6* and plated onto Matrigel containing CCN2. The cells were cultured for 8 h, fixed in 4% PFA, stained with calcein AM, and the number of branch points and tube lengths were evaluated. Two-way ANOVA, *p < 0.05, **p < 0.01, ***p < 0.001. (**G**) LECs were transfected with siRNA against *Dusp6* and proliferation assay was performed.

Time-course experiments showed that phospho-ERK was significantly increased in LECs at 15 min after treatment with CCN2 and continued to increase up to 4 h, followed by a decrease at 8 h (Fig. 5B). The phosphorylation of ERK induced by CCN2 was non-persistent and transient, suggesting the presence of a concomitant mechanism for suppressing ERK. We hypothesized that DUSPs might regulate ERK activation in LECs. Quantitative RT-PCR revealed that *Dusp1, -2, -4, -5, -6, -7, -8, -9* and -*10* are expressed in LECs; *Dusp6* was predominant, but *Dusp16* was undetected (Fig. 6A).

Binding of activated ERK to the kinase interaction motif of DUSP6 results in a conformational change, prompting phosphatase activation of the DUSP6 catalytic domain, leading to dephosphorylation of ERK^26^. To investigate whether ERK and DUSP6 directly interact, we performed immunoprecipitation assays. The results showed that DUSP6 co-precipitated with ERK, and their interaction was enhanced by CCN2 (Fig. 6B). We then examined the subcellular localization of ERK and DUSP6 in CCN2-stimulated LECs by immunostaining. 15 min after CCN2 stimulation, ERK migrated to the nucleus, while DUSP6 localized in the cytoplasm around the nucleus and did not migrate to the nucleus regardless of CCN2 stimulation (Supplementary Fig. S6).

The protein expression level of DUSP6 increased from 12 to 48 h when pERK induced by CCN2 decreased (Fig. 6C). In addition, in LECs transfected with siRNA targeting *Dusp6* (Fig. 6D), phospho-ERK levels were increased compared with levels in cells transfected with non-targeting siRNA at 0.25, 4, 24, and 48 h after CCN2 treatment (*p* = 0.002, *p* < 0.001 for 0.25 h; *p* < 0.001 for 4 h; *p* = 0.007, *p* < 0.001 for 24 h; and *p* = 0.005, *p* < 0.001 for 48 h) (Fig. 6E). The roles of DUSP6 in lymphangiogenesis were evaluated in an in vitro tube formation assay. Similar to the results of in vivo plug assay, CCN2 enhanced tube formation compared with control, such as branch points and tube length per area. When DUSP6-knockdown LECs were stimulated with CCN2, tube formation was increased compared to control cells with CCN2. However, suppression of DUSP6 did not promote tube formation in control gels without CCN2 (Fig. 6F). Next, we examined whether suppression of DUSP6 affected cell proliferation. CCN2 weakly inhibited the cell proliferation of LECs, and DUSP6 suppression further inhibited proliferation, but the effect was minor (Fig. 6G). These results indicate that CCN2 plays a role in promoting tube formation but not cell proliferation in LECs, and that DUSP6 negatively regulates tube formation caused by CCN2.

**Figure 7:**
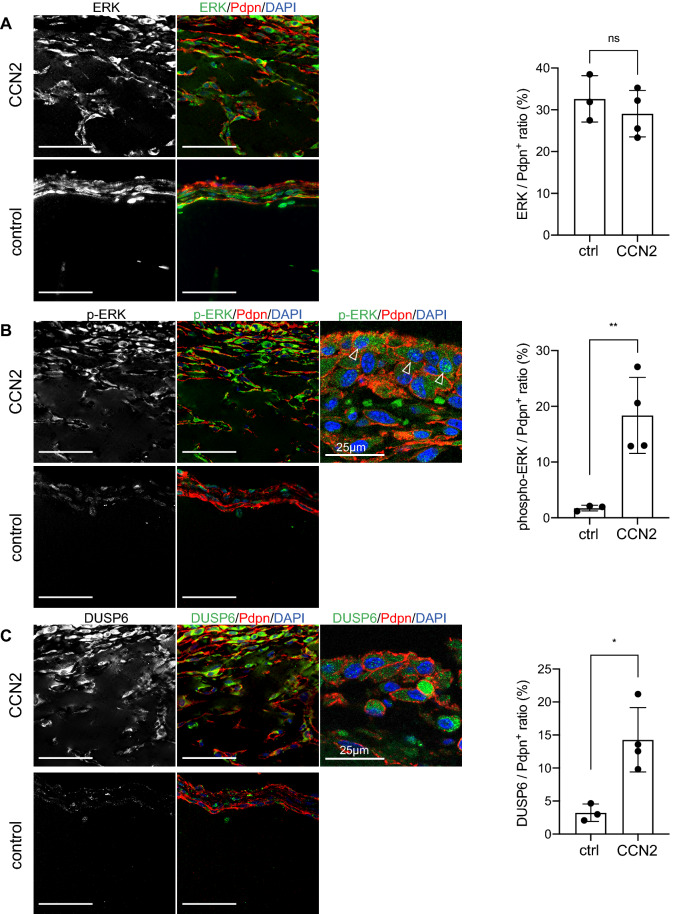
Enhanced expression of phospho-ERK and DUSP6 in the CCN2 Matrigel. Expression of phospho-ERK, DUSP6 and ERK in Matrigel plugs detected by immunostaining (left) and quantification of the ratio of the ERK, phospho-ERK or DUSP6-positive area to the Podoplanin-positive area for each gel (right). ERK (**A**), Phospho-ERK (**B**) and DUSP6 (**C**)-positive cells were detected in both the CCN2 gel and control gel. Arrowheads indicate Podoplanin-positive/phospho-ERK-positive cells. Scale bars: 50 µm. Student *t* test, *p < 0.05, **p < 0.01. ns; not significant.

Immunostaining of Matrigel revealed that total ERK was similarly expressed in both the control gel and the CCN2 gel; however, while phosphorylated ERK and DUSP6 were barely present in the control gel, they were detected in the CCN2 gel and localized in the nucleus and cytosol of Podoplanin-positive cells (Fig. 7A-C). Phosphorylated ERK and DUSP6 expression in Podoplanin-positive cells were significantly increased in the CCN2 gel compared with the control gel (*p* < 0.05) (Fig. 7B,C).

## Discussion

Here, we provide novel insights on the role of CCN2 in lymphangiogenesis. Our results showed that CCN2, a well-known angiogenic factor, promotes lymphangiogenesis in in vivo Matrigel assays, similar to findings in fibrosis model studies^38^. CCN2 also enhanced angiogenesis and peripheral nerve cell migration. Previous angiogenesis studies showed that CCN2 is sufficient to induce angiogenesis, directly or indirectly, under in vitro and in vivo experimental conditions^27,28^. CCN2 expression increases after central nervous system trauma in rodent models^43–45^. In zebrafish spinal cord injury models, injury-induced ccn2a directs glial cell bridging and neuronal regeneration^46^. Our results are consistent with these previous studies.

CCN2 increased the mRNA expression of lymphatic vessel markers but did not change Podoplanin and VEGFR3 protein expression, while LYVE-1 was rather decreased. LYVE-1 in LECs is inactive by default because it is O-terminally glycosylated, and the glycan chain prevents binding to hyaluronan^47^. Because the LYVE-1 detected by Western blotting had a high molecular weight, it could be the glycosyl-modified inactivated form of LYVE-1; CCN2 may decrease the inactivated form of LYVE-1. The molecular mechanism underlying the cell proliferation of LECs involves the activation of ERK signaling stimulated by autocrine or paracrine VEGF-C and VEGFR3 signaling^48^. However, the phosphorylation of VEGFR2 and VEGFR3 was not increased by CCN2, which suggests that CCN2 and endogenous VEGF-C secreted from LECs do not activate VEGF receptor signaling upstream of ERK in primary cultured LECs.

Integrins play key roles in endothelial cell migration and survival during angiogenesis and lymphangiogenesis. Among them, integrins αvβ3 and αvβ5 are both expressed in vascular endothelial cells and promote different angiogenesis pathways^49^. Integrin αvβ3 enhanced angiogenesis which is induced by basic fibroblast growth factors and tumor necrosis factor α, and integrin αvβ5 enhanced VEGF-induced angiogenesis and tumor metastasis^49^. In the present study, both integrin αvβ3 and integrin αvβ5 were detected in LECs, and the phosphorylation of ERK by CCN2 was impaired by antibodies targeting integrins αvβ3 and αvβ5, especially integrin αvβ5. We showed for the first time that integrin αvβ5 is involved in CCN2 signaling in LECs. Integrin α9β1 is required for the proper development of the lymphatic system^49^, while integrin β1 plays an important role in the invasion of LECs^50^. In this study, we also founded that the mRNA expression of *Itga9* and *Itgb1* was high in LECs, but suppression of integrin α9 and β1 did not decrease CCN2-induced ERK phosphorylation. These results indicate that CCN2-induced phospho-ERK is partially mediated by integrins αvβ5 and αvβ3 in LECs. Furthermore, inhibition of HSPGs by heparinase I decreased phosphorylation of ERK by CCN2, suggesting that HSPGs are required for the interaction of CCN2 and integrins αvβ5 and αvβ3 in CCN2-mediated lymphangiogenesis.

The process of lymphangiogenesis in the adult body is based on cell proliferation, branching and migration. Our in vitro cell proliferation assay results showed that CCN2 did not increase the proliferation of LECs but rather weakly suppressed proliferation. In HUVECs, DUSP1 and DUSP5 regulate the MAPK signaling pathways that are activated by VEGF^51^, and DUSP5 modulates ERK1/2 downstream of the Ang1/Tie2 receptors^52^. We showed DUSP6 is highly expressed in LECs, and immunoprecipitation assays showed that CCN2 enhanced the interaction of ERK and DUSP6 in LECs. Suppression of *Dusp6* by siRNA increased phospho-ERK levels compared with control cells. Similar to the in vivo findings, CCN2 promoted tube formation in an in vitro tube formation assay, and suppression of DUSP6 further enhanced tube formation. However, in control gels without CCN2, suppression of DUSP6 did not promote tube formation. Moreover, in the proliferation assay, suppression of DUSP6 did not affect the proliferation of LECs. These results suggest that DUSP6 does not contribute to cell proliferation but inhibits tube formation in CCN2-mediated lymphangiogenesis. In addition, because the effect of DUSP6 occurred only when LECs were stimulated with CCN2, it is assumed that DUSP6 acts as a brake to inhibit abnormal lymphangiogenesis in inflammatory and fibrotic conditions where CCN2 is in excess in vivo.

In immunocytochemistry, both ERK and DUSP6 were detected in the cytoplasm of LECs, and the initial nuclear translocation of phospho-ERK by CCN2 was not impaired. In Matrigel plug assay, phosphorylated ERK and DUSP6 expression were detected in Podoplanin-positive cells in the CCN2 gel. In this study, CCN2-induced ERK signaling peaked within a short period of time, but then persisted to some extent for 4 to 8 h after CCN2 stimulation. Although integrin-mediated MAPK signaling is generally transient, previous reports have shown that CCN2 stimulation sustained ERK activation for at least 9 h in fibroblasts^17^. DUSP6 was increased between 8 and 12 h after CCN2 stimulation, suggesting that the negative regulation of ERK by DUSP6 may be one of the mechanisms underlying the sustained activation of MAPKs by CCN2. In a previous study, Forkhead box C1 (FOXC1) and Forkhead box C2 (FOXC2) were identified as negative regulators of ERK^53^. In mice, lymphatic endothelial cell-specific deletion of FOXC1, FOXC2, or both resulted in lymphatic hyperplasia via hyperactivation of ERK^53^. Here we identified DUSP6 as a suppressor of ERK in LECs; however, the relationship between FOXC1, FOXC2 and DUSP6 has not yet been analyzed.

The discrepancy observed between the in vitro cell proliferation and in vivo Matrigel assays may be due to effects of ECM and MMP in Matrigel. Since CCN2-signaling is mediated by ECM and activated through proteolytic processing by MMP-2, 3^54–56^, lymphangiogenesis in in vivo Matrigel assays was promoted. The results between these assays may not be comparable, and our present results are not contradictory.

A recent study found significant expression of CCN2 in the epithelium and connective tissue of oral submucous fibrosis, which was classified as a potentially malignant oral disease, as well as oral squamous cell carcinoma (OSCC) cases, increasing with disease progression, whereas healthy buccal mucosa had no CCN2 expression^57^. Moreover, mesenchymal stem cell-derived CCN2 promoted tongue squamous cell carcinoma progression in vitro and in vivo. CCN2 is expressed at higher levels in tongue squamous cell carcinoma tissues than in paraneoplastic tissues, and its expression correlated with lymph node metastasis^58^. A study of patient-derived tumor samples in the Cancer Genome Atlas database showed that *CCN2* expression was higher in head and neck cancers, and high expression of *CCN2* and *MMP3* was associated with worse prognosis in head and neck cancers^54^. Lysine-specific demethylase 1 (LSD1) is a nuclear histone demethylase that functions as an epigenetic regulator to promote cancer initiation, progression, and relapse. LSD1 inhibitors markedly downregulated *CCN2* mRNA and protein expression and inhibited further xenograft growth in a tonsillar OSCC patient-derived xenograft mouse model^59^.

We demonstrated that CCN2 activates ERK signaling via integrin αvβ5 and αvβ3 to promote lymphangiogenesis. In addition, CCN2 enhances the binding of DUSP6 to ERK, and DUSP6 suppresses excessive CCN2-induced lymphangiogenesis. In OSCC, CCN2-signaling is associated with disease progression and lymph node metastasis. Our results suggest that integrins αvβ5 and αvβ3, ERK, and DUSP6-mediated lymphangiogenesis may be therapeutic targets of OSCC.

## Methods

### Cell culture and proliferation assay

C57BL/6 mouse primary cultured LECs and VECs (CellBiologics, Chicago, IL, USA) were cultured in 0.1% gelatin-coated culture dishes at 37 °C with 5% CO_2_. LECs were cultured using the EGM2-Endothelial cell growth medium-2 bullet kit (Lonza, Basel, Switzerland). Cells up to 6 passages were used in in vitro experiments.

Cell proliferation was assessed by crystal violet staining method. Cells were plated in 24-well plates at 2 × 10^4^ cells per well and cultured overnight. The medium was changed to medium containing 0, 10, 50 or 100 ng/mL recombinant rat CCN2/CTGF protein, Carrier Free (R&D Systems, Minneapolis, MN, USA). At 0, 24, 48 or 72 h after treatment, cells were fixed with methanol, stained with 0.5% crystal violet (Merck, Darmstadt, Germany) in 25% methanol for 5 min, washed with water five times and dried. Absorbance at 590 nm was measured using the SPARK 10 M plate reader (TECAN, Mannedorf, Switzerland). Cell proliferation was expressed as the ratio of the absorbance of each time point to the absorbance at 0 h.

### Quantitative RT-PCR

Total RNA was prepared from cells using the Purelink RNA purification Kit (Invitrogen, Carlsbad, CA, USA). cDNA was prepared from 1 μg of total RNA using the QuantiTect RT Kit (Qiagen, Venlo, the Netherlands). Quantitative RT-PCR was performed using TB Green Premix ExTaq II (Takarabio, Otsu, Japan) and a LightCycler96 (Roche Basel, Switzerland). Primers used for qPCR are listed in Supplementary Table S1.

### Gene silencing by siRNA

Expression of integrins and DUSP6 was suppressed by siRNA. siRNAs were designed by an siRNA design site, siDirect ver.2 (http://sidirect2.rnai.jp/). Sense and complementary strand RNAs having a two-mer overhang were synthesized and annealed. The siRNA sequences for each mRNA target are listed in Table S2. LECs (1.5 × 10^5^) were transfected with 25 pmol of target siRNA or non-target siRNA (MISSION siRNA universal control#1, Merck) using Lipofectamine RNAiMAX (Thermo Fisher, Waltham, MA, USA) according to the manufacturer’s instructions.

### Western blot analysis

LECs were pre-cultured with FBS-free and supplement-free EGM2 medium (Lonza) for 24 h. Cells were then treated with CCN2-containing FBS-free and supplement-free EGM2 medium and cultured for the appropriate time. Cells were washed with PBS and lysed in RIPA Buffer (Merck) containing cOmplete protease inhibitor cocktail (Roche) and phosphatase inhibitor cocktail (Nacalai, Kyoto, Japan). Protein concentration was determined with the BCA protein assay kit (Thermo). Protein samples (5–20 µg) were separated with SDS-PAGE and transferred to PVDF membranes (Invitrogen). The membranes were blocked in Blocking-one (Nacalai) for 30 min and cut prior to hybridization with antibodies, then incubated with primary antibodies at 4 °C overnight. The primary antibodies are listed in Supplementary Table S3. After three washes with PBS containing 0.1% Tween-20, the membrane was incubated with HRP-conjugated anti-rabbit IgG or HRP-conjugated anti-rat IgG secondary antibody (Cell Signaling Technology, Danvers, MA, USA) for 2 h. After three washes with PBS containing 0.1% Tween-20, signals were visualized using ECL Prime (GE Healthcare, Chicago, IL, USA) and an image analyzer 680 (GE Healthcare). Signals were quantified using ImageQuant TL 8.1 software (GE Healthcare).

### Phospho-receptor tyrosine kinase array

LECs were pre-cultured with FBS-free and supplement-free basal medium for 24 h and then treated with medium containing 100 ng/mL of recombinant rat CCN2/CTGF (R&D Systems) for 5 min. The phosphorylation levels of RTK were analyzed using a Proteome profiler mouse phospho-RTK array kit (R&D Systems) according to the manufacturer’s instructions. Briefly, 200 µg of total protein was incubated with the receptor antibody-spotted membrane at 4 °C for 24 h. After washing, the membrane was incubated with anti-phospho tyrosine kinase antibody, and the phosphorylation levels of receptor tyrosine kinase were detected with the image analyzer 680 (GE Healthcare).

### Immunoprecipitation assay

Cells were lysed in Lysis Buffer (CST) containing cOmplete protease inhibitor cocktail and phosphatase inhibitor cocktail. Protein concentration was determined with the BCA protein assay kit (Thermo). Cell lysates containing 200 µg of total protein were incubated with anti-ERK (CST) or anti-DUSP6 (Abcam, Cambridge, UK) antibody at 4 °C overnight. Protein A magnetic beads (New England Biolabs, Ipswich, MA, USA) were then added to the cell lysate and antibody mixture, and the samples were mixed for 20 min. Magnetic beads were separated and washed with lysis buffer five times; the samples were denatured in 20 µl of 3 × SDS Sample Buffer and subjected to Western blotting.

### Immunocytochemistry

To study the phosphorylation of ERK and the localization of DUSP6, LECs were plated in 35 mm glass bottom dishes (Iwaki, Tokyo, Japan), pre-cultured with FBS-free and supplement-free basal medium for 24 h and then treated with medium containing 100 ng/mL of recombinant rat CCN2/CTGF (R&D Systems) for 0, 5, 15, 30, 60, 120 min. Cells were fixed in 4% paraformaldehyde in PBS. Cell permeabilization was performed by using ice-cold 100% methanol, incubate in methanol for 20 min at − 20 °C. After washing three times with PBS, cells were blocked in Blocking-one Histo (Nacalai) for 30 min and then incubated with primary antibodies at 4˚C overnight. The primary antibodies are listed in Supplementary Table S3. After washing three times with PBS, cells were incubated for 30 min with secondary antibodies: Alexa488 donkey anti-mouse IgG and Alexa594 donkey anti-rabbit IgG (Jackson Immunolaboratory, West Crove, PA, USA). DAPI (4,6-diamino-2-phenyl indole) was used as a nuclear counterstain. Images were acquired using a confocal microscopy (LSM710; Carl Zeiss, Tokyo, Japan). To examine integrin expression, LECs were plated on glass bottom dishes and incubated overnight, followed by immunostaining using anti-integrin αvβ3 (LM609) and anti-integrin αvβ5 (P1F6) antibodies (Abcam). LECs were fixed by 4% paraformaldehyde in PBS and then blocked with PBS containing 1% BSA for 1 h. The primary antibody was diluted in PBS containing 1% BSA and the antibody reaction was performed overnight at 4 °C. After the cells were washed with PBS, a secondary antibody reaction was performed using Alexa488 donkey anti-mouse IgG. Actin and nuclei were stained with Alexa Fluor plus 405 Phalloidin (Thermo Fisher Scientific, Tokyo, Japan) and 7-AAD (7-Aminoactinomycin D) (BD Biosciences, Tokyo, Japan) and observed by fluorescence microscopy (BZ-710X; Keyence, Kyoto, Japan).

### Tube formation assay

A Matrigel-based tube formation assay was performed as previously described. LECs were transfected with *Dusp6* siRNA or non-targeting siRNA. Each well of a 24-well plate was coated with 100 μL growth factor-reduced Matrigel (Corning, Corning, NY, USA) with or without 1.6 μg CCN2, which was allowed to polymerize for 30 min at 37 °C. Then, LECs were seeded onto the coated wells at a density of 5 × 10^4^ cells per well and cultured in 500 μL FBS-free and supplement-free EGM2 medium. After incubation for 8 h at 37 °C under 5% CO_2_, tube formation images were captured at 100 × magnification by microscopy (BZ-710X; Keyence) in three random fields. For quantification, the tube length and branching points were measured.

### In vivo* Matrigel plug assay*

Matrigel plug assay was conducted to measure the lymphangiogenesis as previously described^60^. Male 8-week-old C57BL/6 mice were purchased from Charles River Japan. Mice were kept under specific pathogen-free conditions and used at 9 weeks of age. All experiments were performed in compliance with the relevant laws and institutional guidelines and were approved by the Animal Care and Use Committee of Fukuoka University (approval number: 2008024), and in accordance with the ARRIVE guidelines.

We mixed 450 µL of growth factor reduced Matrigel (Corning) and 8.33 µg of recombinant rat CCN2/CTGF (R&D Systems) in 50 µL of PBS or 50 µL of PBS as control. We then randomly divided into two groups (CCN2; n = 4, control; n = 3), and subcutaneously injected the Matrigel mixture on the back of each mouse under anesthesia by 2% isoflurane. At day 7 after injection, mice were euthanized with cervical dislocation. Matrigel plugs were removed, fixed in 4% paraformaldehyde in PBS, and embedded in paraffin. Sections (4 µm thick) were deparaffinized and stained with hematoxylin–eosin (HE). Primary antibodies used for immunohistochemistry are listed in Supplementary Table S3. The following secondary antibodies were used: Alexa594 goat anti-Syrian hamster IgG, Alexa488 donkey anti-rat IgG and Alexa488 donkey anti-rabbit IgG (Jackson). Morphology was observed using a fluorescence microscopy (BZ-710X; Keyence), and samples were examined using a confocal microscope (LSM710; Carl Zeiss).

### Statistical analysis

Statistical analysis was performed using GraphPad Prism software version 8. All experiments were performed more than three times, except the RTK assay (n = 2), and data are expressed as the mean ± standard error (S.E.). Comparative analysis was performed by Student’s *t* test, one-way and repeated measures analysis of variance (ANOVA), or two-way ANOVA. For multiple comparisons, we performed Bonferroni or Sidak analysis. Statistical significance was set at a *p*-value of less than 0.05.

## Supplementary Information

Supplementary Information.

## Data Availability

All data are contained within the article.
