# Supplementary material for: CCN2-induced lymphangiogenesis is mediated by the integrin αvβ5–ERK pathway and regulated by DUSP6
Source: Sci Rep. 2022 Jan 18;12:926. doi: 10.1038/s41598-022-04988-4 (PMC8766563; doi:10.1038/s41598-022-04988-4)

CCN2-induced lymphangiogenesis is mediated by the integrin $\alpha v \beta 5$-ERK pathway and regulated by DUSP6

Shiho Hashiguchi[1, 2†], Tomoko Tanaka[2†], Ryosuke Mano[1, 2], Seiji Kondo[1], Shohta Kodama[2*]

[1] Department of Oral Surgery, Faculty of Medicine, Fukuoka University

[2] Department of Regenerative Medicine and Transplantation, Faculty of Medicine, Fukuoka University

Supplementary Information

Supplementary Table S1. List of primers used for quantitative RT-PCR.

Target	GenBank Acc.	Sense 5'->3'	Antisense 5'->3'
Actb	NM_007393.5	CATCCGTAAAGACCTCTATGCCA	ATGGAGCCACCGATCCACA
Lyve1	NM_053247.4	AGCAGCCTTGTTGGCTGAGA	CTGTCTACGTGCTGCAGATCAATTA
Podoplanin	NM_001290822.1	GGCTTAATGAATCTACTGGCAAG	ATCATGGTCTGAGGTTGCTGAGG
Prox1	NM_001360827.1	CTTGCCCACTCTTGGAGCAC	CATATGCAAATGAGCCATTCCTG
Vegfc	NM_009506.2	CAGTGCATGAACACCAGCACA	TAGACATGCACCGGCAGGAA
Vegfd	NM_001308489.1	TGCTGTGGGATAACACCAAATGTAA	AGACGCACTCACAGCGATCTTC
Kdr	NM_001363216.1	ACAGACAGTGGGATGGTCCTTG	GTAGCCACTGGTCTGGTTGGAG
Flt4	NM_008029.3	GCAGGACAGGCGACCATACA	AGCTTGACACTCAATCCCACAGAC
Pecam1	NM_008816.3	ATGAGGACCAGTCCCCGAAG	ACTCCAATGACAACCACCGC
Itgav	NM_008402.3	GGAACTCAACTGCTCGCTGG	ACGGCGAGGTCAACCTTGTA
Itga9	NM_001113514.1	GCTGTCTGGGAGGAGGCTAA	GTCTGGCCCTGAGGAGAACC
Itga2b	NM_010575.2	CATGGAAGCGTGTCCATCGT	CTGAGGTCGAAGAGCAACGG
Itgb1	NM_010578.2	CCATGCCAGGGACTGACAGA	GAGCTTGATTCCAATGGTCCAGA
Itgb2	NM_008404.5	GCATCTGTGGGCAGTGTGTA	ATTTGCCACAGTTGCAGGA
Itgb3	NM_016780.2	GAAGTTCAACCGGGGAACGC	TGCCAGTATCCGTCAGCTCT
Itgb5	NM_001145884.1	AAGATTGGGGACACGGCATC	CGGGCCTCAAGGTGAAAGAC
Dusp1	NM_013642.3	CTTCTTCGCTTTCAACGCCG	TCAGCGTTGGGCACGATATG
Dusp2	NM_010090.2	CCTGCTGGGGCCGAAAATAG	TGCAGGCCCTGAAGATCTGA
Dusp4	NM_176933.4	CTGGCAGTGAACCAGCACTC	GTACAAGTGCATCCCCGTCG
Dusp5	NM_001085390.2	CCCGCGGGTCTACTTCCTTA	AGGCTTCTCTCGCCTTCGAT
Dusp6	NM_026268.3	TCTGTTTGAGAATGCGGGCG	CCAAGCAATGCACCAGGACA
Dusp7	NM_153459.4	GCCAGGCCTACTACCTCCAA	AGGACGAGCTGTCCACGTTA
Dusp8	NM_008748.3	CAGGGCAAGCGAGAGCC	GAGTGACCCCTGAAGTGAGGA
Dusp9	NM_029352.3	TACGACCAGGGTAGCAGTGG	CCGCTTCGTTCTCCGCTTC
Dusp10	NM_022019.6	GTGTTGCTGCTGGGGATCAAT	GAGGAGGCTGAAAACTGGCAA
Dusp16	NM_001048054.2	CACCCTTCAGAGGAGAAGCCC	GCCCTCACTCCAACAGCTTTA

Supplementary Table S2. List of siRNA sequences.

Target gene	Sequence 5'->3'	Complementary strand 5'->3'
Itgav#1	AAUGAUAUUAAAAUCUAAGUC	CUUAGAUUUUAAUAUCAUUUA
Itgav#2	AAUUCUAUGAUAUUAAAGGAA	CCUUUAAUAUCAUAGAAUUCC
Itga9#1	AAAUAUGAAAGAUACAAAGAU	CUUUGUAUCUUUCAUAUUUGG
Itga9#2	UUGAUAAAACUUAAAAGAGCU	CUCUUUUAAGUUUUAUCAAAA
Itgb1#1	UUAAACAUCUAUUUUUAUCUG	GAUAAAAAUAGAUGUUUAAAA
Itgb1#2	UUGUAUAUACAUUAUUUUCCA	GAAAAUAAUGUAUAUACAAUG
Itgb3#1	AUUGUAACAGGGGUUUUUGAU	CAAAAACCCCUGUUACAAUA
Itgb3#2	ACUUUAGAGCGGAUUUUCCCG	GGAAAAUCCGCUCUAAAGUGG
Itgb5#1	AAUAACUUGUAACCAAUACAC	GUAUUGGUUACAAGUUAUUCC
Itgb5#2	AUUGUAACAGGGGUUUUUGAU	CAAAAACCCCUGUUACAAUA
Dusp6#1	AGGAAUUUGCUUGUAUUUGAA	CAAAUACAAGCAAAUUCCUAU
Dusp6#2	UCAUCUAUGAAGAAAUGGCC	CCAUUUCUUUCAUAGAUGAAG

Supplementary Table S3. List of antibodies used for Western blotting, immunoprecipitation assay, immunocytochemistry and immunohistochemistry.

Target	Antibody	Supplier/ catalogue number
phospho-ERK	Phospho-p44/42 MAPK (Erk1/2) (Thr202/Tyr204) Antibody	Cell Signaling Technology / 9101
ERK (for WB)	p44/42 MAPK (Erk1/2) Antibody	Cell Signaling Technology / 9102
ERK (for IP and IHC)	p44/42 MAPK (Erk1/2) (137F5) rabbit mAb	Cell Signaling Technology / 4695
ERK (for ICC)	p44/42 MAPK (Erk1/2) (L34F12) mouse mAb	Cell Signaling Technology / 4696
phospho-p38	Phospho-p38 MAPK (Thr180/Tyr182) (3D7) rabbit mAb	Cell Signaling Technology / 9215
p38	p38 MAPK Antibody	Cell Signaling Technology / 9212
phosho-JNK	Phospho-SAPK/JNK (Thr183/Tyr185) Antibody	Cell Signaling Technology / 9251
JNK	SAPK/JNK Antibody	Cell Signaling Technology / 9252
phospho-AKT	Phospho-Akt (Ser473) (D9E) XP rabbit mAb	Cell Signaling Technology / 4060
AKT	Akt (pan) (C67E7) rabbit mAb	Cell Signaling Technology / 4691
phospho-NFκB	Phospho-NF-κB p65 (Ser536) (93H1) rabbit mAb	Cell Signaling Technology / 3033
NFκB	NF-κB p65 (D14E12) XP rabbit mAb	Cell Signaling Technology / 8242
HSP90	HSP90 Antibody	Cell Signaling Technology / 4874
GAPDH	GAPDH (14C10) rabbit mAb	Cell Signaling Technology / 2118
phospho-Smad1/5/9	Phospho-Smad1 (Ser463/465)/ Smad5 (Ser463/465)/ Smad9 (Ser465/467) (D5B10) rabbit mAb	Cell Signaling Technology / 13820
Smad1	Smad1 (D59D7) XP rabbit mAb	Cell Signaling Technology / 6944
Smad5	Smad5 (D4G2) rabbit mAb	Cell Signaling Technology /12534
phospho-Smad2	Phospho-Smad2 (Ser465/467) (138D4) rabbit mAb	Cell Signaling Technology / 3108
Smad2	Smad2 (D43B4) XP rabbit mAb	Cell Signaling Technology / 5339
phospho-Smad3	Phospho-Smad3 (Ser423/425) (C25A9) rabbit mAb	Cell Signaling Technology / 9520
Smad3	Smad3 (C67H9) rabbit mAb	Cell Signaling Technology /9523
Integrin αv	Anti-Integrin alpha V antibody [EPR16800]	abcam / ab179475
Integrin α9	Anti-Integrin alpha 9 antibody [EPR9722]	abcam / ab140599
Integrin β1	Anti-Integrin β1 Antibody, clone MB1.2	R&S systems / MAB1997

Integrin β3	Anti-Integrin beta 3 antibody [ERP17507]	abcam / ab179473
Integrin β5	Mouse/Rat Integrin beta 5 Antibody	R&S systems /AF8035
Integrin αVβ3	Anti-Integrin alpha V beta 3 antibody [LM609]	abcam / ab190147
Integrin αVβ5	Anti-Integrin alpha V+beta 5 antibody [P1F6]	abcam / ab1777004
DUSP6	Anti-DUSP6 antibody [EPR129Y]	abcam / ab76310
LYVE-1	Rabbit Anti-Mouse Lyve-1	Relia Tech / 103-PA50AG
Podoplanin	Purified anti mouse Podoplanin Antibody	Biolegend / 127401
CD31	Anti-Pecam-1 Antibody, clone 390	Merck / CBL1337
β3-Tubulin	β3-Tubulin (D65A4) XP rabbit mAb	Cell Signaling Technology / 5666

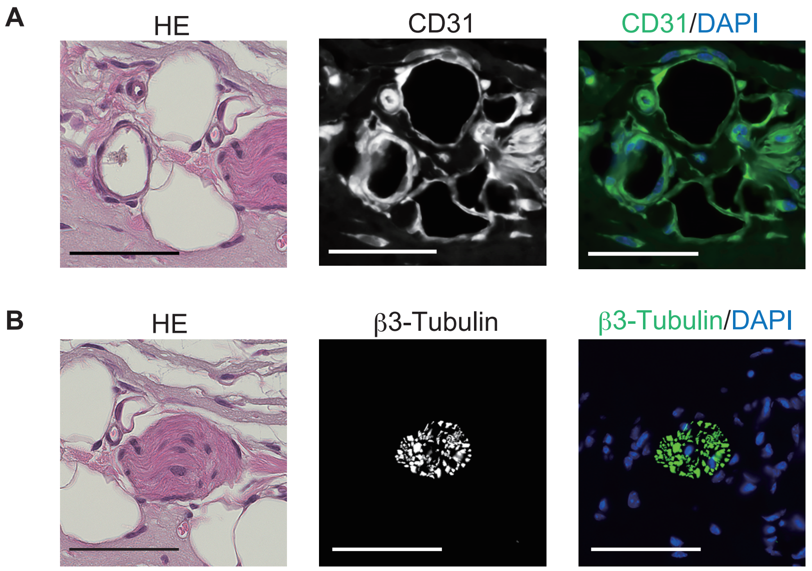

Supplementary Figure S1. CD31 positive or β3-Tubulin positive tubular structure was induced by CCN2 in Matrigel plug. Matrigel and recombinant CCN2 were mixed and injected subcutaneously back of the mice. Control mice was injected with Matrigel and PBS mixture. Seven days after injection, Matrigel plug was removed, and expression of CD31 and β3-Tubulin was assessed by immunohistochemistry. (A) CD31 positive vessels were detected in CCN2 plug. (B) β3-Tubulin positive bundles detected in CCN2 plug. Scale bars: 50 μm.

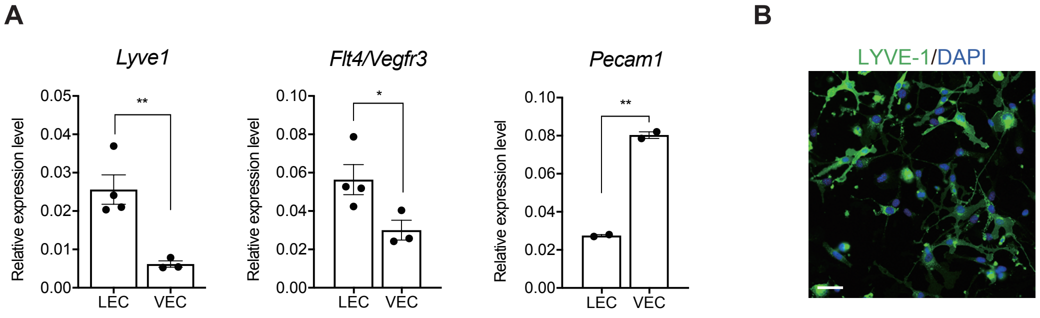

Supplementary Figure S2. Characterization of LECs (A) Expression levels of *Lyve1* and *Vegfr3*, lymphatic vessel markers, and *Pecam1*, a vascular marker, in mouse primary cultured LECs and vascular endothelial cells (VEC) were analyzed with quantitative RT-PCR. Two-way ANOVA, $*p < 0.05$, $**p < 0.01$ (B) Immunocytochemistry of LYVE-1 in mouse primary cultured LECs . Scale bars: 50 μm.

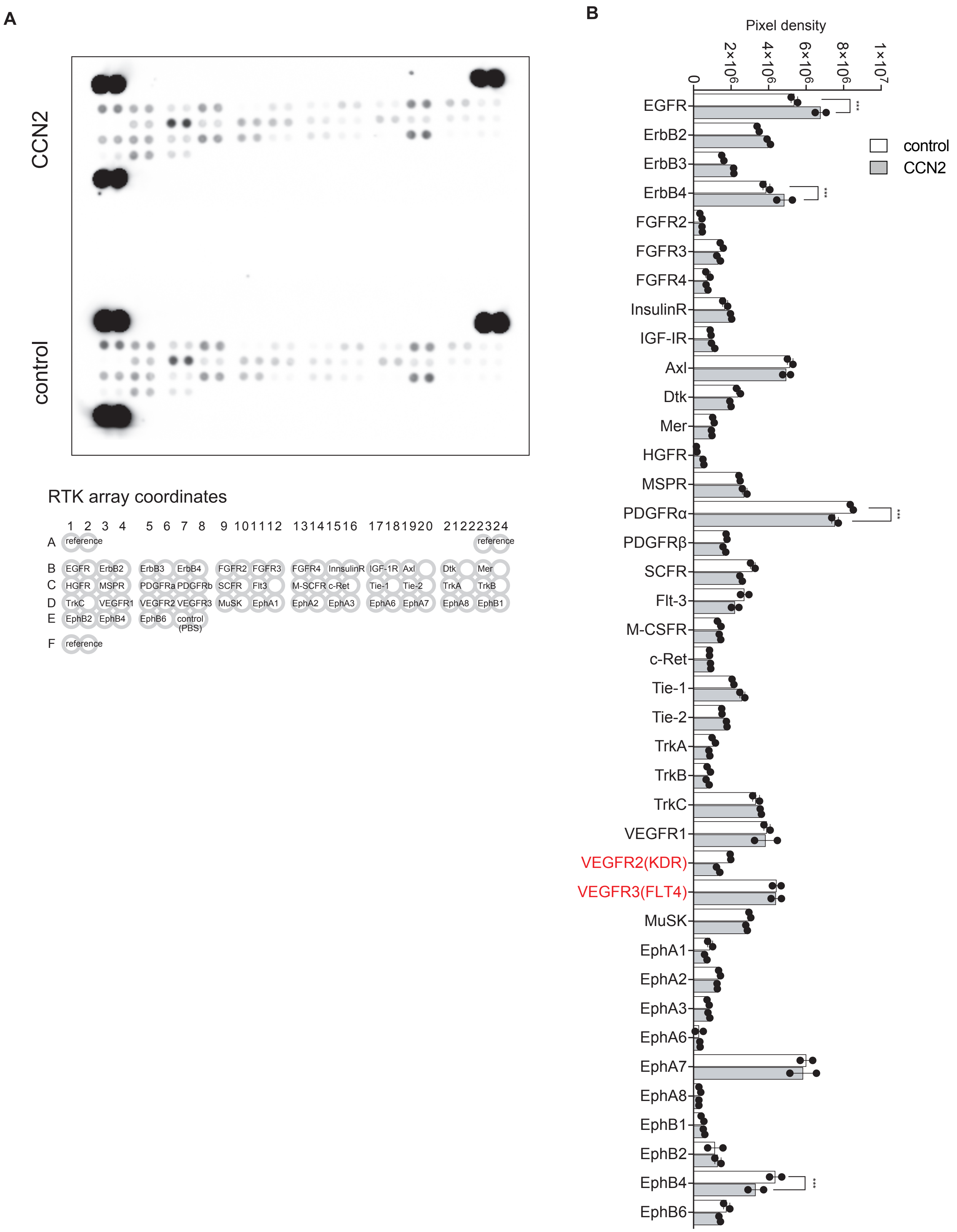

Supplementary Figure S3. Phospho-RTK array. Cell lysate containing 200 μg of protein prepared from LECs treated with 100 ng/mL CCN2 or PBS (as control) for 15 min was incubated with antibody-spotted membrane at 4˚C overnight. Membrane was washed with wash buffer, incubated with anti-phospho-tyrosine antibody for 2hr. (A) Phosphorylated RTK was visualized with a image analyzer. (B) Pixel density of each RTK was measured by ImageQuant TL 8.1 software, and subtracted by that of reference. Two-way ANOVA, ***p < 0.001.

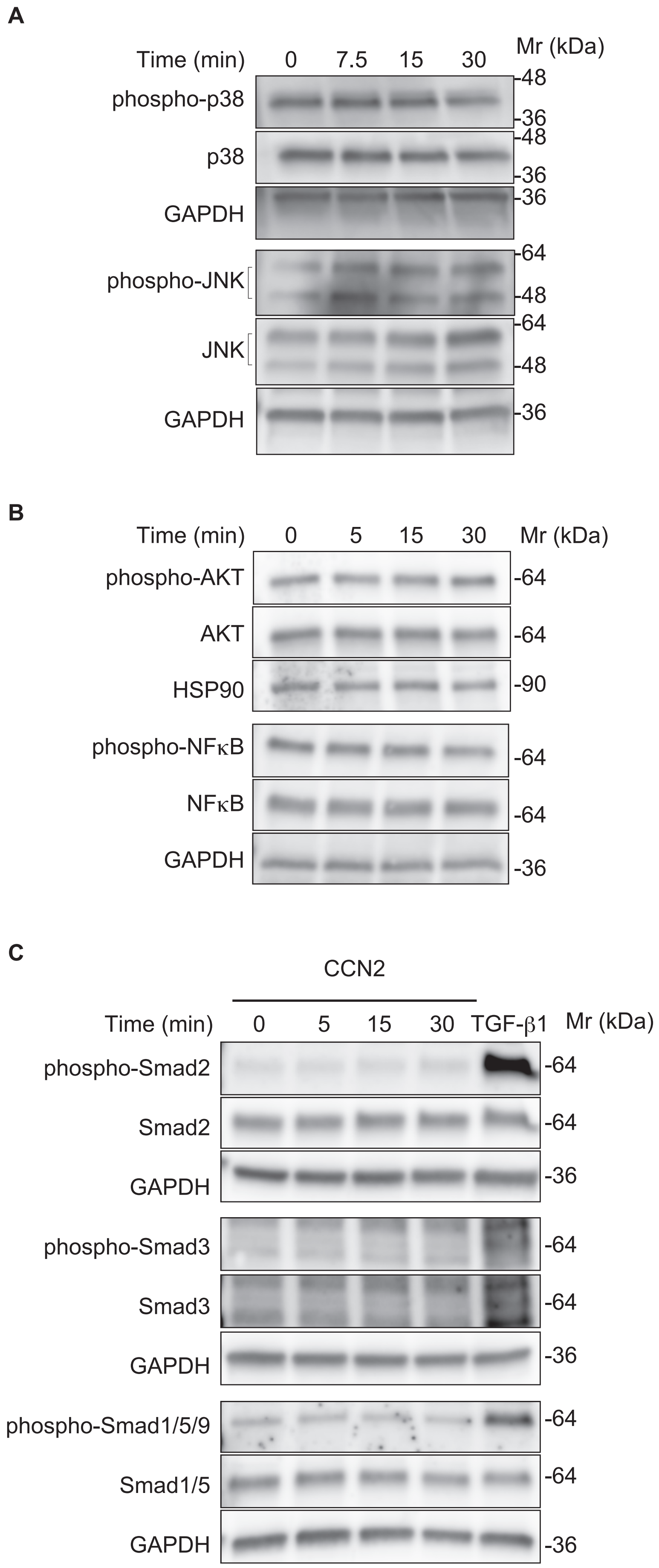

Supplementary Figure S4. Phosphorylation levels of p38 MAPK, JNK, AKT, NFkB, Smad2, 3, 1/5/9 in LECs treated with CCN2. LECs were treated with 100 ng/mL of CCN2 for 0, 5 or 7.5, 10 and 30 min and lysed in RIPA buffer. 5-10 µg of total protein was subjected to Western blotting. (A) phospho-p38 and phospho-JNK, (B) phospho-AKT and phospho-NFκB, (C) phospho-Smad2, phospho-Smad3 and phospho-Smad1/5/9. Cell lysate of LECs treated with TGFβ1 for 30 min was used for control for phospho-Smad3.

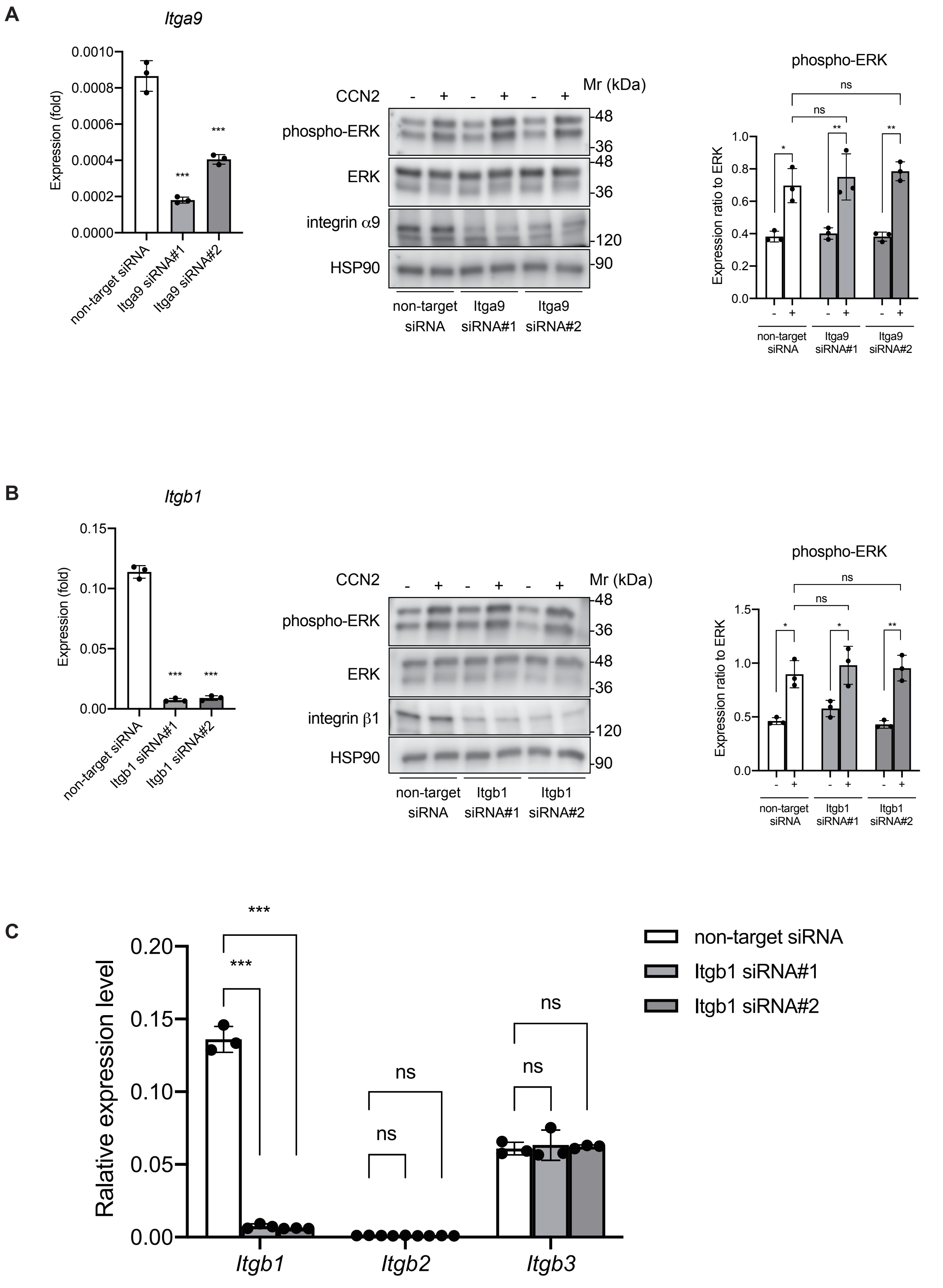

Supplementary Figure S5. ERK phosphorylation in LECs under suppression of Itga9 or Itgb1 expression.
LECs were transfected with siRNA for *Itga9*, *Itgb1* or non-target siRNA, cultured for 24 hour, and stimulated with 100 ng/mL CCN2, and phospho-ERK levels of LECs suppressed with Itga9 (A) and Itgb1 (B) were detected by Western blot. (C) Expression levels of *Itgb2* and *Itgb3* in Itgb1-suppressed LECs were analyzed with quantitative RT-PCR. Two-way ANOVA, $*p < 0.05$, $**p < 0.01$, $***p < 0.001$. ns; not significant.

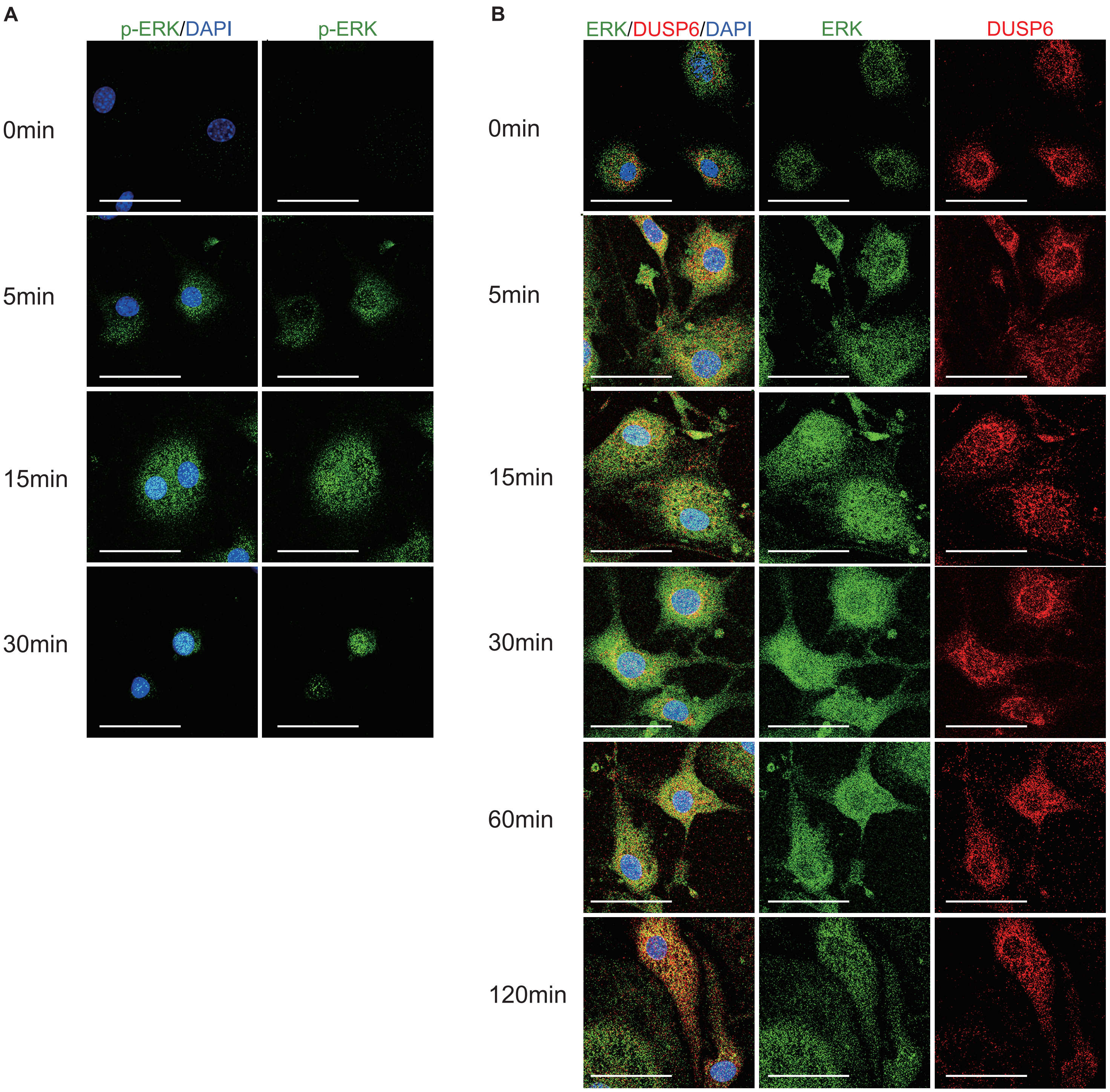

Supplementary Figure S6. DUSP6 is involved in the dephosphorylation of cytoplasmic ERK in LECs. LECs were treated with 100 ng/mL of CCN2 for 0, 5, 15, 30, 60, and 120 min. Expression of phospho-ERK (A), DUSP6 and ERK (B) in LECs were detected by immunostaining. Scale bars: 30 μm.

The images of the original blots

Fig2. C

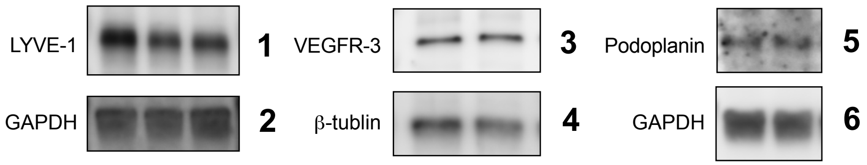

Original images

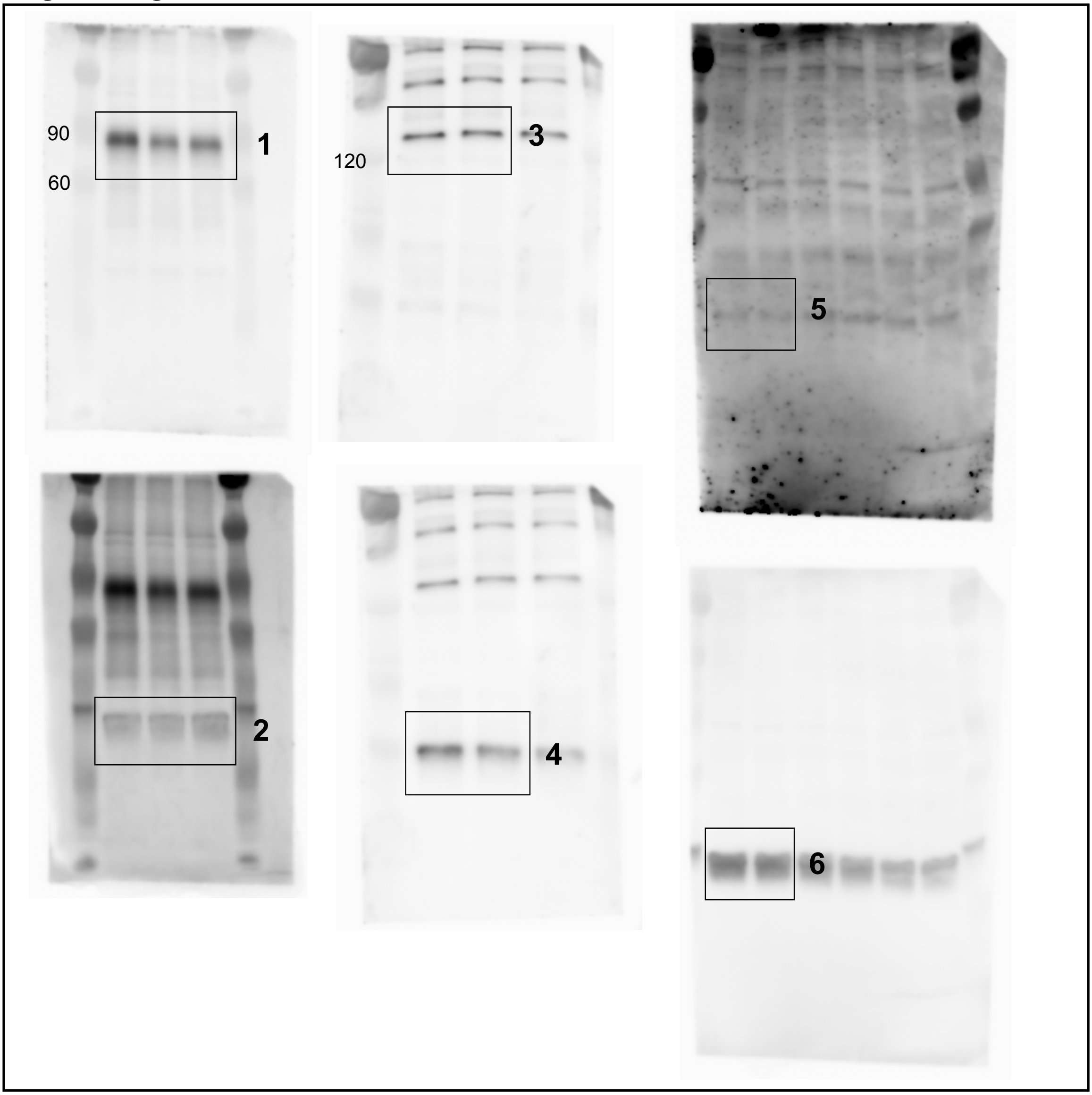

Fig3.A

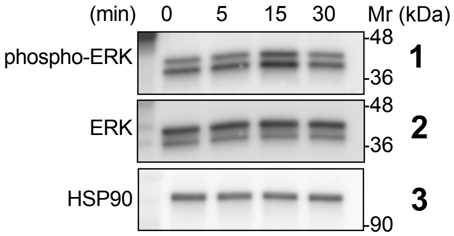

Original images

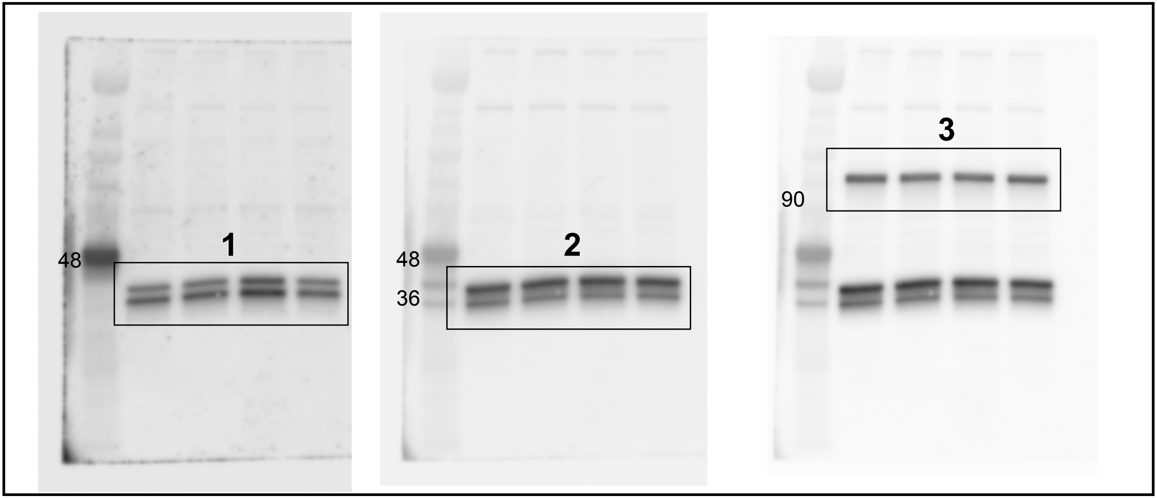

Fig4. D

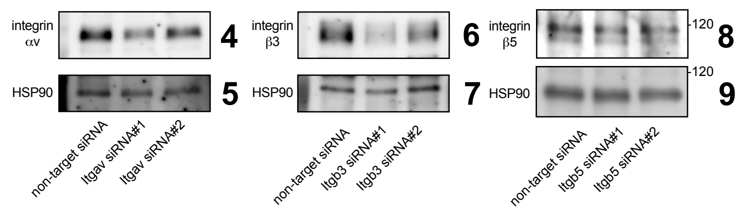

Fig4. E

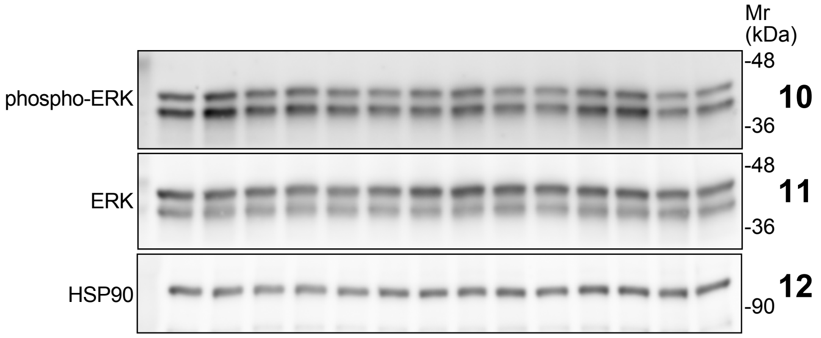

Fig4. F

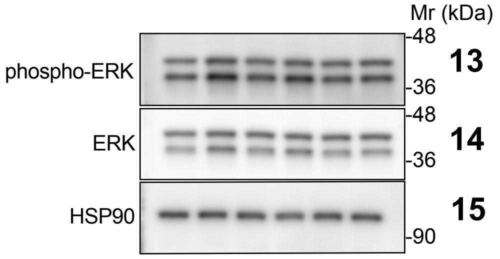

Fig4. G

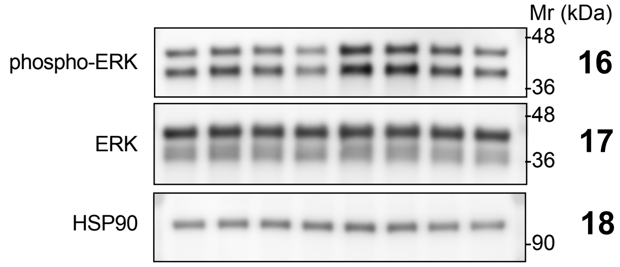

Original images

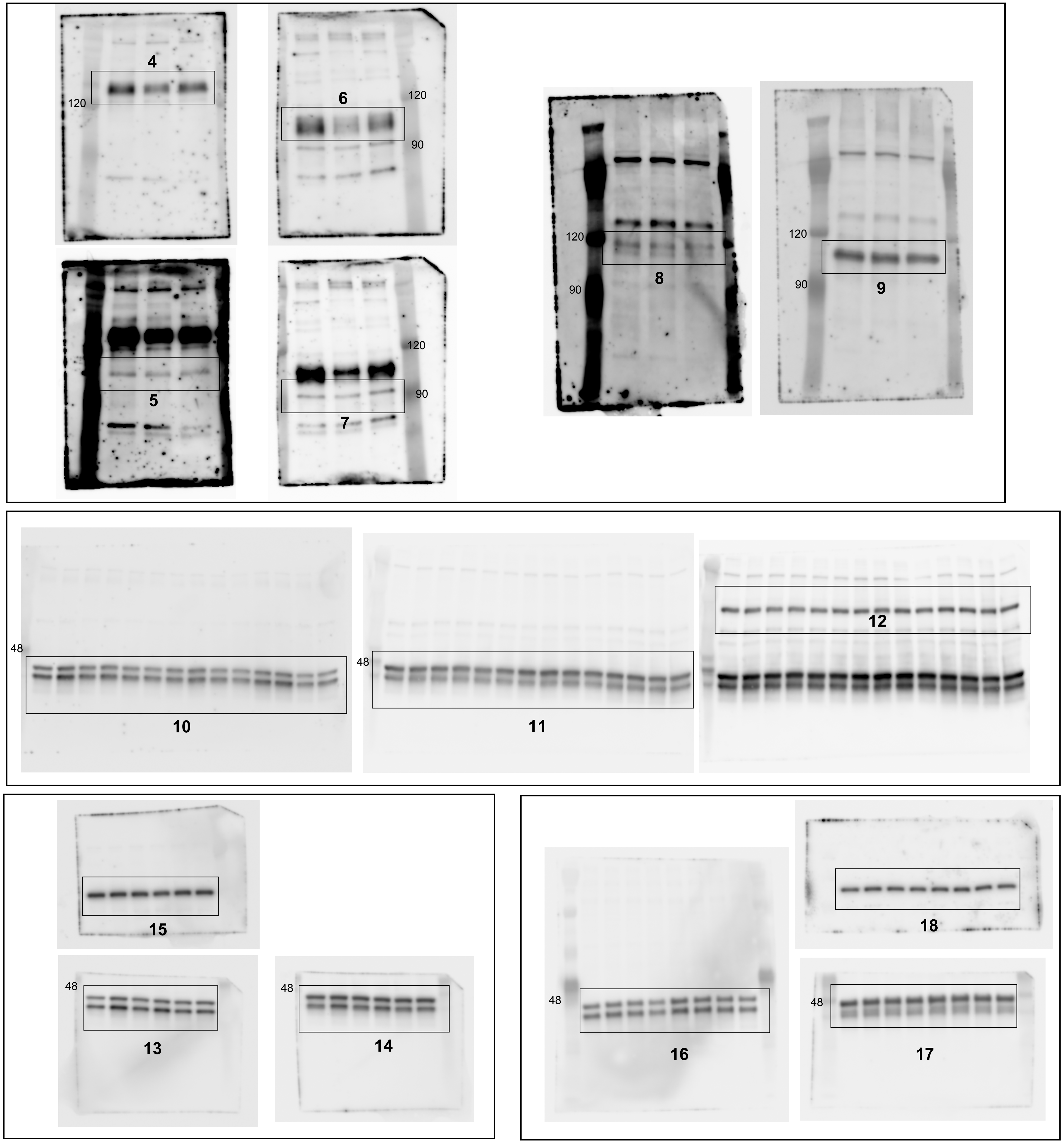

Fig5.B

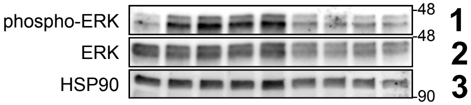

Original images

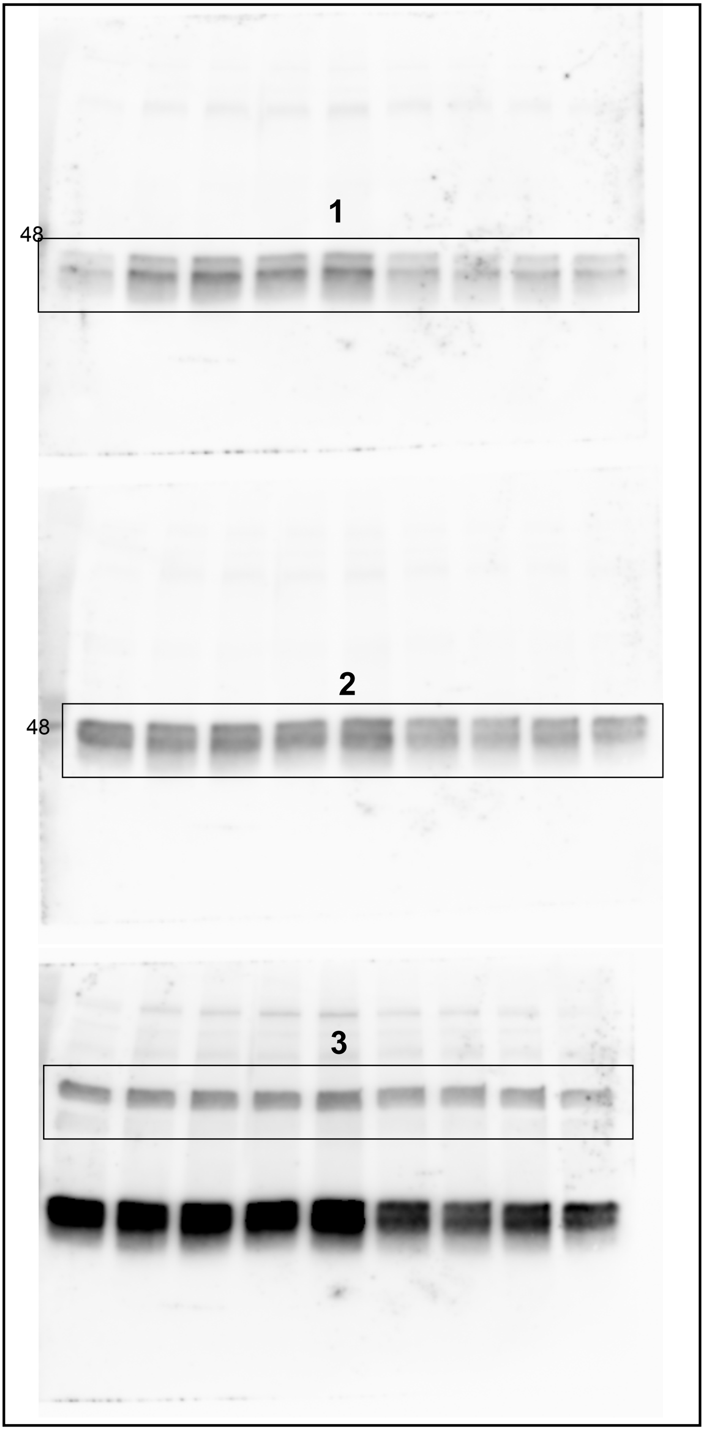

Original images

Fig6.B

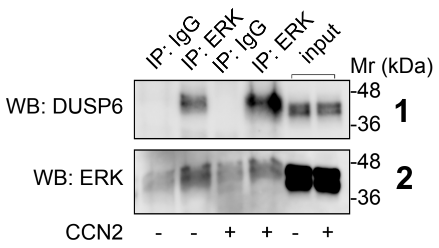

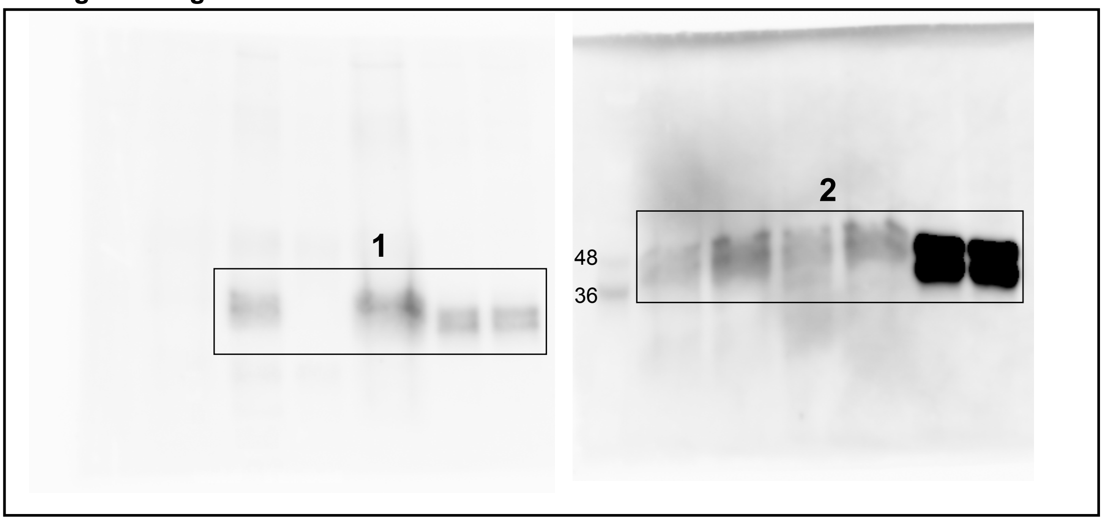

Fig6.C

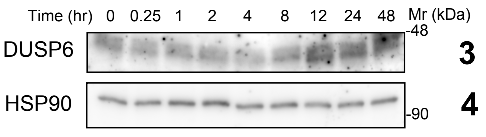

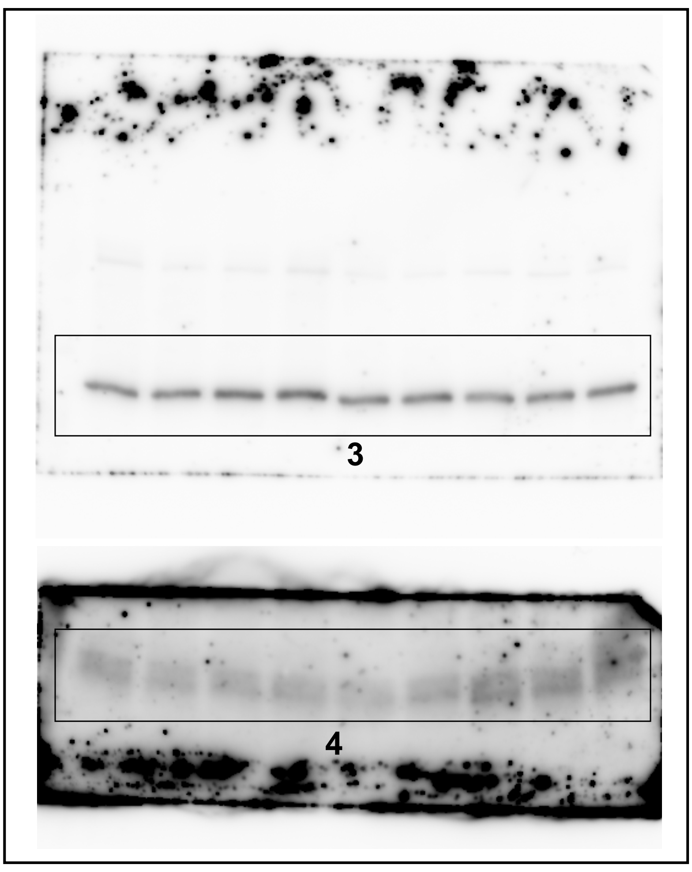

Fig6.D

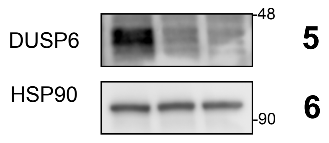

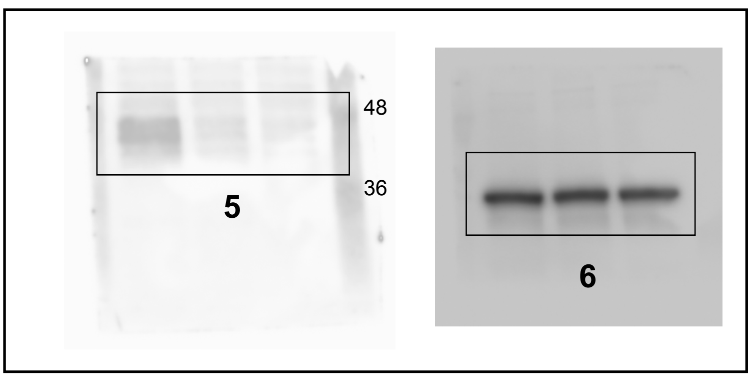

Fig6.E

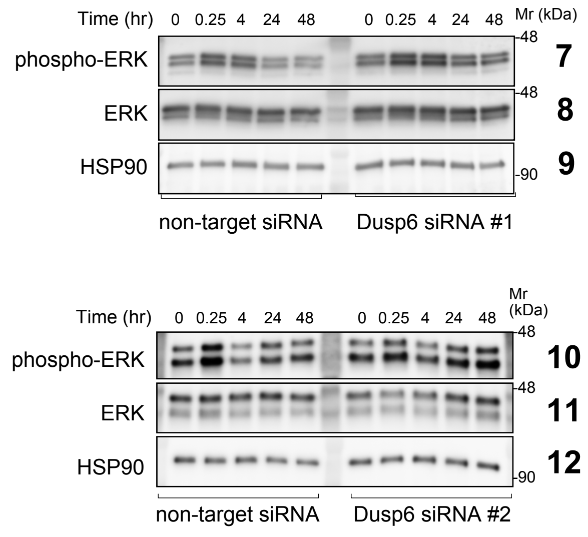

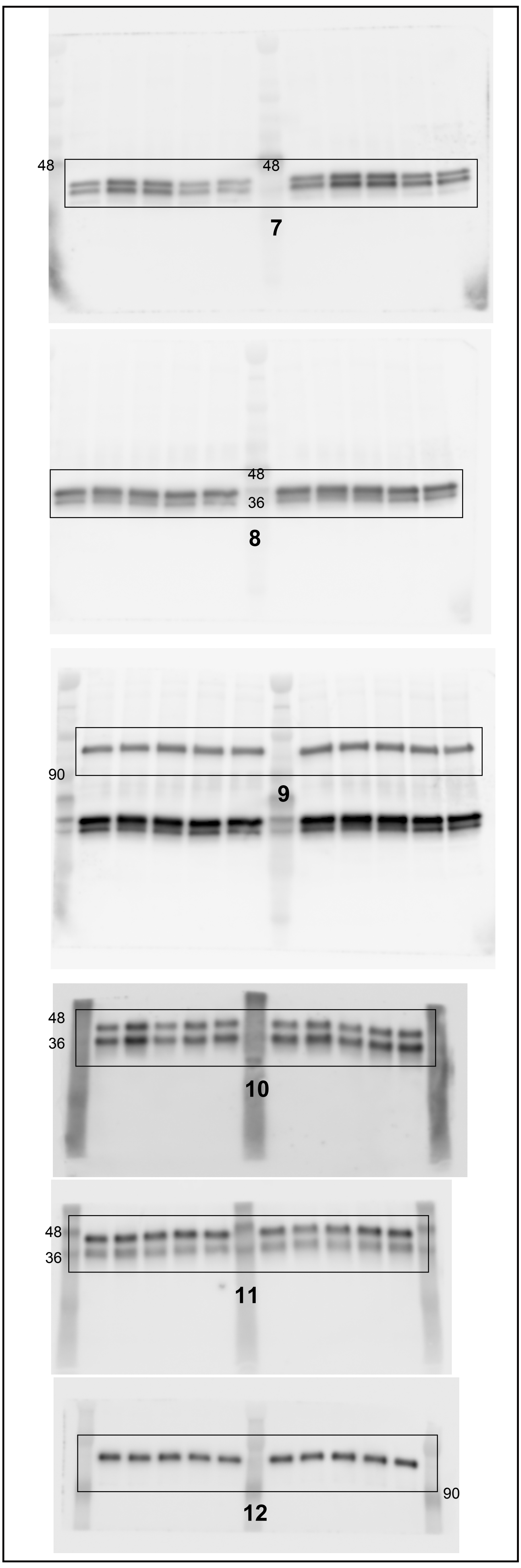

Supplementary Figure S4.A

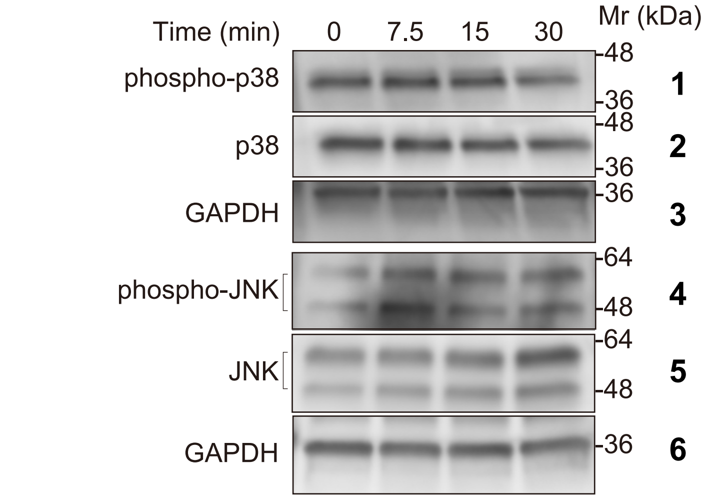

Original images

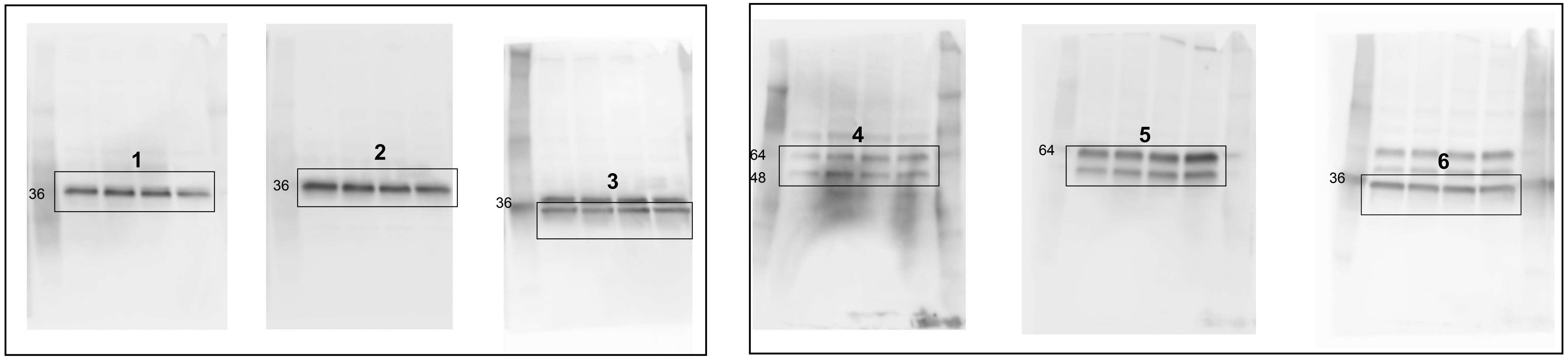

Supplementary Figure S4.B

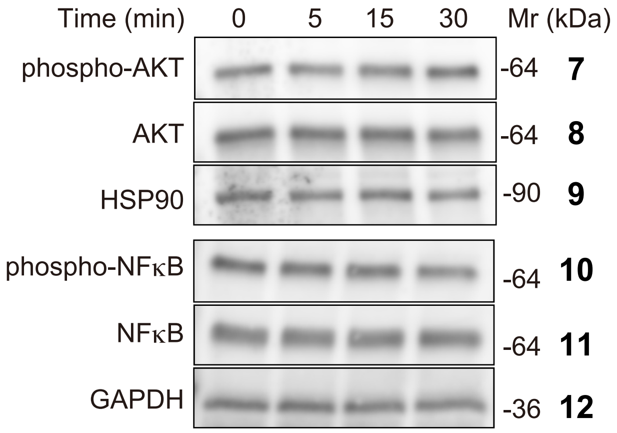

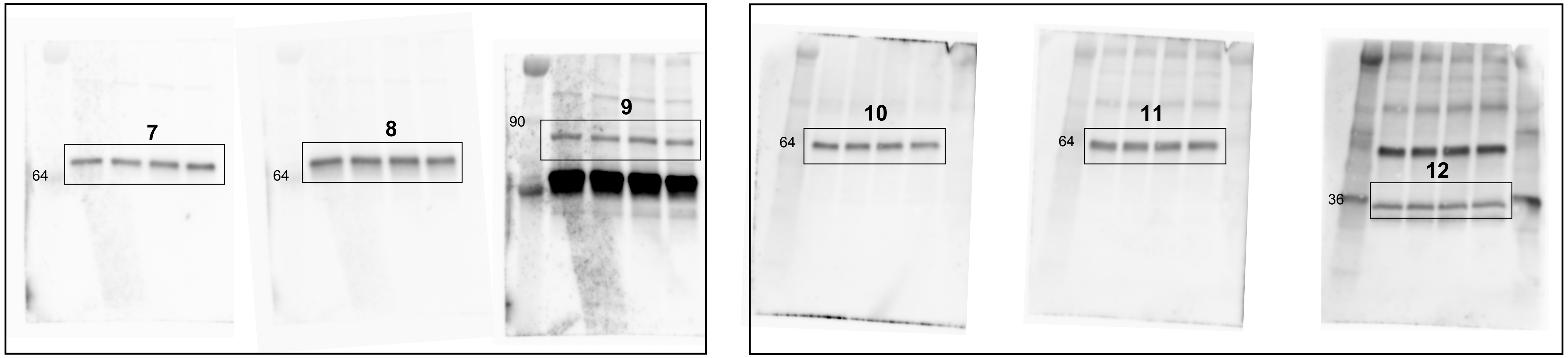

Supplementary Figure S4.C

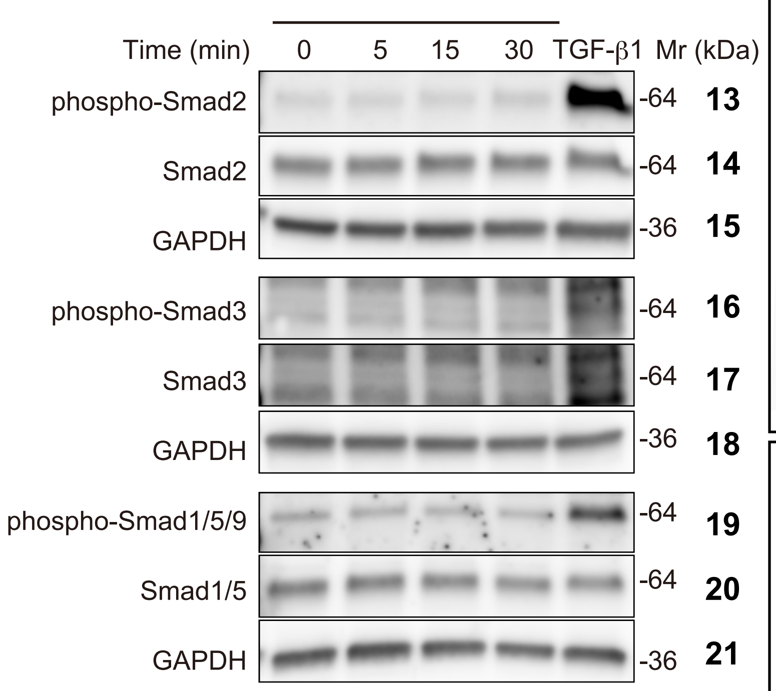

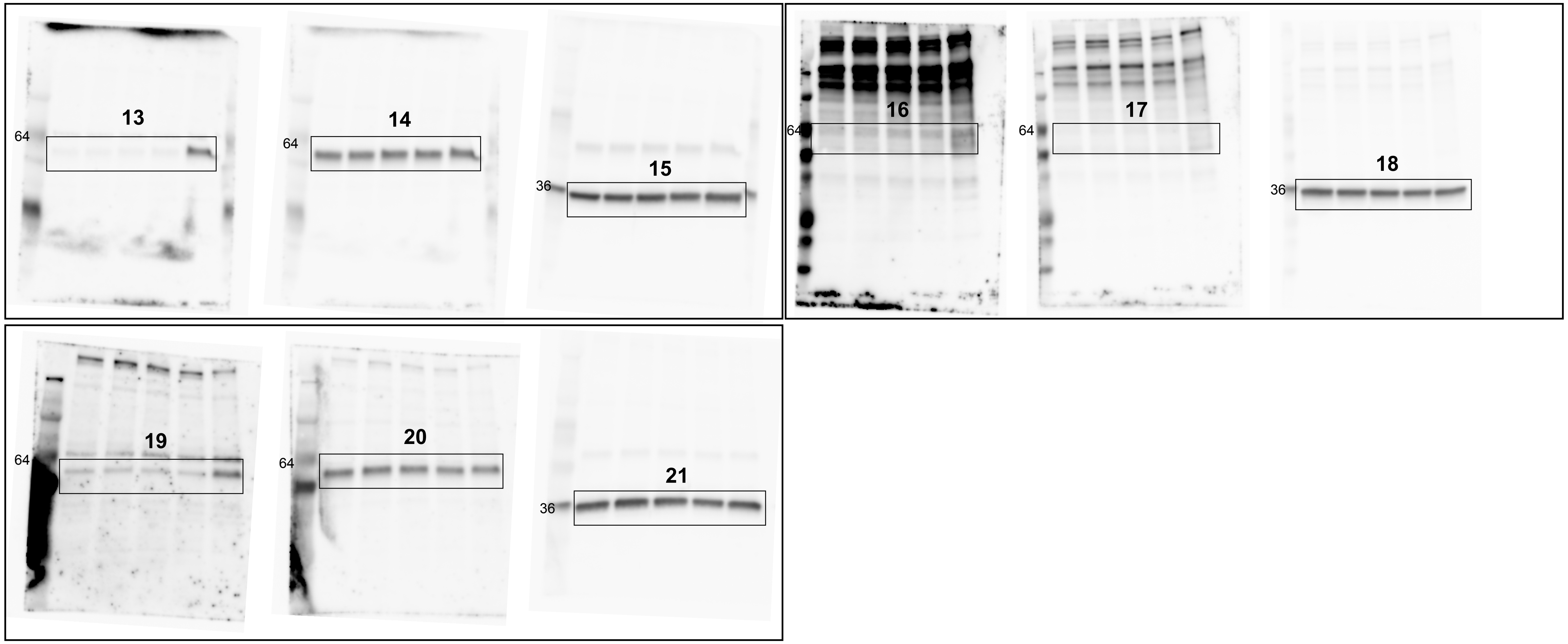

Supplementary Figure S5. A

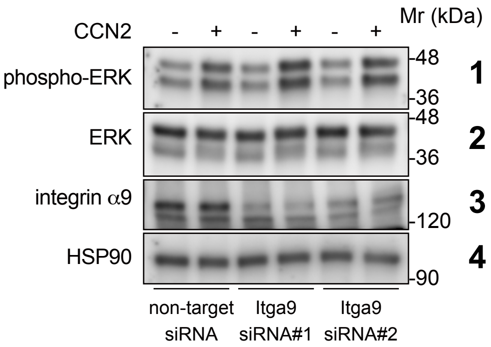

Supplementary Figure S5. B

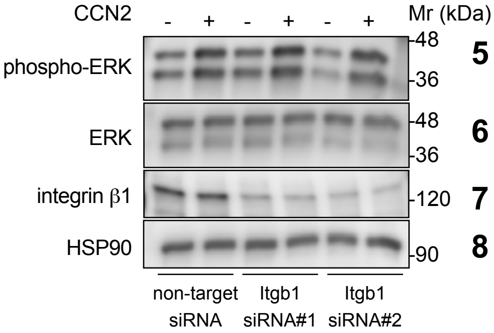

Original images

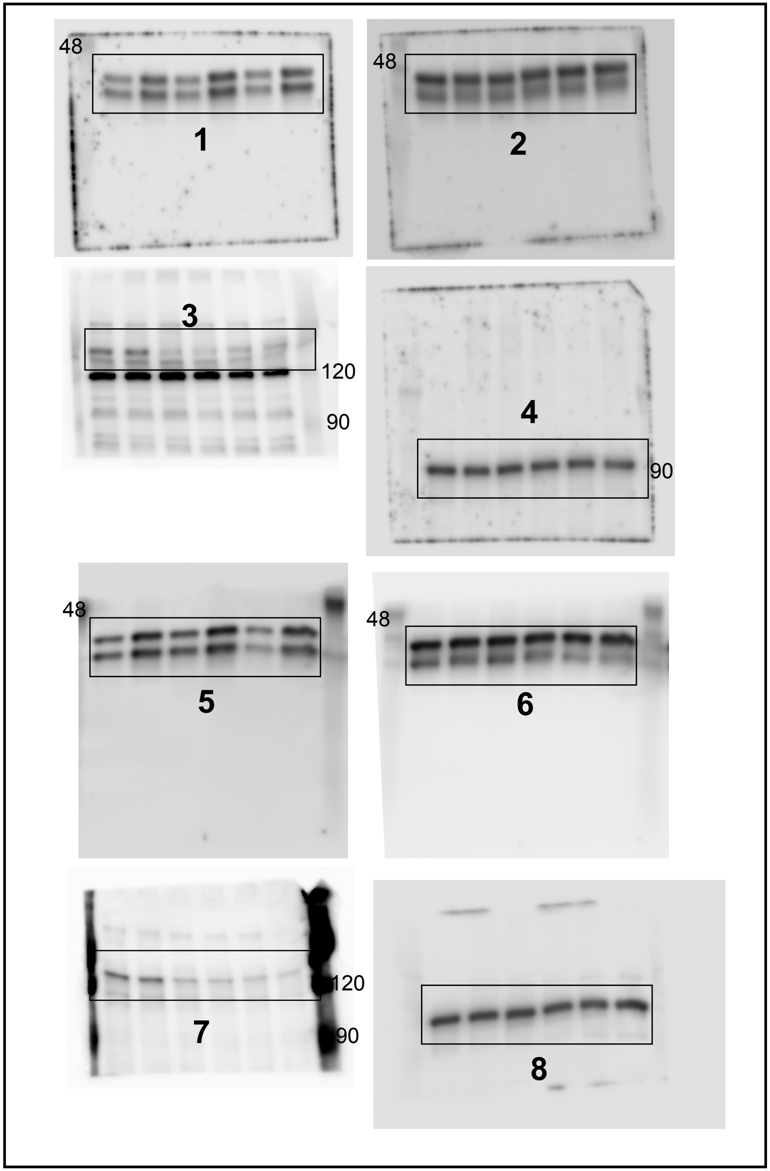

Supplement: Supplementary file 1 — Supplementary Information. [file 41598_2022_4988_MOESM1_ESM.pdf]
